# Reactive Oxygen Species Derived from NOX3 and NOX5 Drive Differentiation of Human Oligodendrocytes

**DOI:** 10.3389/fncel.2016.00146

**Published:** 2016-06-02

**Authors:** Roberta Accetta, Simona Damiano, Annalisa Morano, Paolo Mondola, Roberto Paternò, Enrico V. Avvedimento, Mariarosaria Santillo

**Affiliations:** ^1^Dipartimento di Medicina Clinica e Chirurgia, Università degli Studi di Napoli Federico IINaples, Italy; ^2^Laboratori di Ricerca Preclinica e Traslazionale, Istituto di Ricovero e Cura a Carattere Scientifico – Centro di Riferimento Oncologico della BasilicataRionero in Vulture, Italy; ^3^Dipartimento di Medicina Molecolare e Biotecnologie Mediche, Università degli Studi di Napoli Federico IINaples, Italy

**Keywords:** NOX3, NOX5, reactive oxygen species, oligodendrocyte, differentiation, multiple sclerosis

## Abstract

Reactive oxygen species (ROS) are signaling molecules that mediate stress response, apoptosis, DNA damage, gene expression and differentiation. We report here that differentiation of oligodendrocytes (OLs), the myelin forming cells in the CNS, is driven by ROS. To dissect the OL differentiation pathway, we used the cell line MO3-13, which display the molecular and cellular features of OL precursors. These cells exposed 1–4 days to low levels of H_2_O_2_ or to the protein kinase C (PKC) activator, phorbol-12-*Myristate*-13-Acetate (PMA) increased the expression of specific OL differentiation markers: the specific nuclear factor Olig-2, and Myelin Basic Protein (MBP), which was processed and accumulated selectively in membranes. The induction of differentiation genes was associated with the activation of ERK1-2 and phosphorylation of the nuclear cAMP responsive element binding protein 1 (CREB). PKC mediates ROS-induced differentiation because PKC depletion or bis-indolyl-maleimide (BIM), a PKC inhibitor, reversed the induction of differentiation markers by H_2_O_2_. H_2_O_2_ and PMA increased the expression of membrane-bound NADPH oxidases, NOX3 and NOX5. Selective depletion of these proteins inhibited differentiation induced by PMA. Furthermore, NOX5 silencing down regulated NOX3 mRNA levels, suggesting that ROS produced by NOX5 up-regulate NOX3 expression. These data unravel an elaborate network of ROS-generating enzymes (NOX5 to NOX3) activated by PKC and necessary for differentiation of OLs. Furthermore, NOX3 and NOX5, as inducers of OL differentiation, represent novel targets for therapies of demyelinating diseases, including multiple sclerosis, associated with impairment of OL differentiation.

## Introduction

Reactive oxygen species (ROS), including hydrogen peroxide (H_2_O_2_), superoxide anion (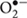) and hydroxyl radical (•OH), are highly reactive molecules generated by the chemical transformation of oxygen inside the cells. High levels of ROS induce cell damage causing oxidation of macromolecules and play an important role in the pathogenesis of many disorders, including inflammatory and neurodegenerative diseases (Maes et al., 2011). However, controlled ROS generation play a physiological role in redox-sensitive gene expression and cell signaling by regulating physiological processes (Sauer et al., 2001; D’Autréaux and Toledano, 2007).

A growing body of evidence links cell differentiation with ROS generation (Burdon and Rice-Evans, 1989; Rao and Berk, 1992). Redox sensitive signaling pathways have also a role in the development of nervous system cells (Morrison, 2001; Kageyama et al., 2005; Kennedy et al., 2012). In addition, in the adult brain the oxygen tension is one of important factor driving the proliferation and differentiation of Neural Stem Cells (NSC) (Panchision, 2009). A direct role of ROS in neuronal differentiation has been demonstrated *in vitro* in PC12 (Katoh et al., 1997) or neuroblastoma cells (Nissen et al., 1973). ROS dependent signaling is also involved in glial cells specification and differentiation (Cavaliere et al., 2012). In oligodendrocyte (OL) a key determinant of the balance between self-renewal and differentiation is the intracellular redox state of precursor cells (Smith et al., 2000). OLs are responsible in CNS for myelin sheath formation assuring the fast saltatory transmission of the action potentials in neurons. In the adult brain, the turnover of OLs and myelination take place continuously ensuring continuous myelin formation. OLs originate from OL precursor cells (OPCs) (Clemente et al., 2013). These cells progress through pre-OLs, immature OLs and mature non-myelinating OLs, before reaching their final stage of post-mitotic myelin forming cells. Each step of maturation is characterized by the expression of specific markers (Baumann and Pham-Dinh, 2001). It is likely that any redox unbalance in OPC or OL may alter terminal differentiation and seriously compromise the formation of myelin forming cells. Multiple sclerosis (MS) is an inflammatory autoimmune disease characterized by multifocal demyelinating lesions in the white matter of CNS. OL replacement and remyelination rarely occur in MS lesions (Franklin and Ffrench-Constant, 2008; Lopez Juarez et al., 2015), suggesting that impaired OL differentiation may represent the ultimate consequence of OL-targeted inflammation. It is known that inflammation leads to the activation of oxidative stress and, as a consequence, high levels ROS can be achieved within MS lesions influencing the local environment where OPCs maturation and remyelination occurs (di Penta et al., 2013).

Our data suggest that maturation of OL is tightly associated to redox balance and the two NOX3 and 5 enzymes seem to be relevant modulators of ROS homeostasis in OL. ROS are generated by different systems such as mitochondrial electron transport chain enzymes, xanthine/xanthine oxidase system and membrane NADPH oxidases (NOXs), which were originally found in phagocytic cells. In mammalian there are seven NOX genes encoding distinct catalytic subunits, namely NOXs 1-5 and DUOX1-2 (Bedard and Krause, 2007). There are several structural and functional differences among NOXs isoforms (Lambeth, 2004). NOX1-4 share a common structure characterized by six *trans*-membrane helices and a short N-terminus. The catalytic subunits of NOX1-4 are associated with the integral membrane protein, p22phox. NOX1-3 require membrane translocation of cytosolic components for their activity. NOX5 is activated by calcium ions. NOXs isoforms are differentially expressed in cells and tissues; in many cases, multiple NOXs are expressed in the same cell type with different subcellular localization and functions (Santillo et al., 2015).

NOX-derived ROS seem to be involved in NMDA-induced OL differentiation in the subventricular region in rat brain (Cavaliere et al., 2013). However, little is known about the modulation of NOX expression during OLs differentiation and the role of the different NOX isoforms. This is relevant because different subcellular localization of NOXs may determine the availability of ROS in specific cell compartments (Li et al., 2006; Oakley et al., 2009; Prosser et al., 2011). However, we wish to note that most of our knowledge about the biology of OLs derives from studies in rodents and therefore the role of NOXs-dependent redox signaling in human model of OL differentiation has not yet been thoroughly addressed.

Here we sought to evaluate the involvement of redox signaling dependent by NOXs isoforms in the molecular mechanisms leading to human OLs differentiation. To this end we evaluated the effects of low levels of H_2_O_2_ and the involvement of the membrane-bound superoxide generating NADPH oxidase NOXs in human OL differentiation.

We report that OL cells MO3-13, with the phenotypic characteristics of human OPCs, express NOX3 and NOX5 isoforms and when exposed to low H_2_O_2_ levels (200 μM H_2_O_2_), express early and late OL differentiation markers. Moreover, OL differentiation, induced by 100 nM Phorbol-12-Myristate-13-Acetate (PMA) in the absence of serum, is dependent on ROS generated by these two NOXs isoforms.

## Materials and Methods

### Cell Cultures

The MO3-13 cells (CELLution Biosystem Inc., Canada) are an immortal human–human hybrid cell line with the phenotypic characteristics of primary OLs, derived from the fusion of a 6-thioguanine-resistant mutant of a human rhabdomyosarcoma with OLs obtained from adult human brain. They were grown in Dulbecco’s Modified Eagles Medium (DMEM; GIBCO Invitrogen), containing 4.5 g/L glucose (GIBCO, Auckland, New Zealand), supplemented with 10% Fetal Bovine Serum (FBS; Sigma S. Louis, USA), 100 U/ml penicillin and 100 μg/ml streptomycin. The cells were kept in a 5% CO_2_ and 95% air atmosphere at 37° C.

### PMA and H_2_O_2_ Cell Treatment

The cells were differentiated in FBS-free DMEM, supplemented with 100 nM of Phorbol-12-Myristate-13-Acetate (PMA; Sigma–Aldrich) until 4 days and the FBS-free medium containing PMA was replaced every day.

To study the effects of chronic H_2_O_2_ treatment on OL differentiation, MO3-13 cells were incubated with 200 μM of H_2_O_2_ until 4 days in complete medium in the absence of any other differentiation stimulus and the medium containing H_2_O_2_ was replaced every day.

When not differentiated cells were compared to 1 or 4 days differentiated cells, the treatments were staggered to allow harvesting all cell samples at the same time.

### Flow Cytometric Assay of Cell Viability

The toxicity of H_2_O_2_ treatment was tested by cytofluorimetric analysis of Propidium Iodide (PI) staining of PMA-differentiated (4 days) and not differentiated MO3-13 cells. Both cell samples, plated in 35 mm Petri dishes, were treated with increasing doses of H_2_O_2_ (200, 400, 600, 800, and 1000 μM) for 18 h in medium without serum. After trypsinization and wash in PBS, the cells were resuspended in 500 μl of PBS and 1 μg/ml of PI was added before the flow cytometric analysis of PI-positive cells performed with a FCSscan apparatus (Becton-Dickinson). Data were analyzed using WinMDI 2.9 software.

### Inhibitors Treatment and PKC Depletion

MO3-13 cells were preincubated for 1 h with 1-(4-Hydroxy-3-methoxyphenyl)ethanone, also known as Apocynin (50 μM, Sigma–Aldrich) or for 30 min with Bisindolylmaleimide VIII acetate (BIM, 100 μM, Sigma–Aldrich), *N*-acetyl Cysteine (NAC, 200 μM, Sigma–Aldrich), Cu,Zn superoxide dismutase (SOD1, 400 ng/ml) and then stimulated for 4 days with PMA (100 nM) or with H_2_O_2_ (200 μM).

Protein kinase C-depleted MO3-13 cells were prepared by treating the cells with PMA 1 μM for 24 h in medium containing 0.2% FBS (Rogers et al., 1990) and then stimulated with H_2_O_2_ (200 μM) for 30min.

### Western Blotting Analysis

MO3-13 cell lysates were obtained in RIPA buffer (50 mM Tris-HCl, pH 7.5, 150 mM NaCl, 1% NP40, 0.5% deoxycholate, 0.1% sodium dodecyl sulphate (SDS) containing 2.5 mM Na-pyrophosphate, 1 mM β-glycerophosphate, 1 mM NaVO4, 1 mM NaF, 0.5 mM phenyl-methyl-sulfonyl-fluoride (PMSF), and a cocktail of protease inhibitors (Roche Applied Bioscience). The cells were kept for 15 min at 4°C and disrupted by repeated aspiration through a 21-gage needle. Cell lysates were centrifuged for 10 min at 11,600 × *g* and the pellets were discarded. Fifty micrograms of total proteins were subjected to SDS – 10% polyacrylamide gel electrophoresis (SDS-PAGE) under reducing conditions. After electrophoresis, the proteins were transferred onto a nitrocellulose filter membrane (GE-Healthcare, Amersham PI, UK) with a Trans-Blot Cell (Bio-Rad Laboratories, Berkeley, CA, USA) and transfer buffer containing 25 mM Tris, 192 mM glycine, 20% methanol. For proteins detection membranes were placed in 5% non-fat milk in tris-buffered saline, 0.1% Tween 20 (TBST, Bio-Rad Laboratories) at room temperature for 2 h to block the non-specific binding sites. Filters were incubated with specific rabbit polyclonal antibodies against Olig-2 (Millipore), p-Creb (Ser 133) (Millipore), NOX3 (abcam), NOX5 (abcam), p-PKCα (Ser657) (Upstate) or a specific mouse polyclonal antibody against p-Erk (Santa Cruz Biotecnology, INC.), and then incubated with a peroxidase-conjugated anti-rabbit or anti-mouse secondary antibody (GE-Healthcare, UK). Peroxidase activity was detected with the enhanced chemiluminescence (ECL) system (GE-Healthcare). To normalize for sample loading and protein transfer the membranes were then stripped and reprobed with an anti α-tubulin antibody (Sigma–Aldrich) or an anti Total Erk 1–2 (Santa Cruz Biotecnology, INC.). Protein bands were revealed by ECL and, when specified, quantified by densitometry using ImageJ software.

### Flow Cytometric Analysis of Myelin Basic Protein (MBP)

Cells were grown to semiconfluency in 60-mm culture dishes. After detachment by trypsin, 5 × 10^5^ cells are suspended in 1 mL of phosphate buffered saline (PBS) and fixed overnight with 1% formaldehyde at room temperature. Next, cells were permeabilized with 0.1% Triton X-100 for 40 min at 4°C, washed 4x with PBS containing 2% FBS, 0.01% NaN_3_, 0.1% Triton X-100 (buffer A), and incubated for 45 min at 4° C with 1:50 dilution of rabbit polyclonal anti-human *MBP* Ig. The cells were then washed twice with the same buffer and incubated for 45 min at 4°C with Cy3-conjugated anti-(rabbit IgG) Ig at 1:50 dilution. Control cells were incubated with Cy3-conjugated anti-(rabbit IgG) Ig alone. After two washes in buffer A, cells were resuspended in PBS and analyzed by flow cytometry using FACSCAN (BD, Heidelberg, Germany) and WINMDI 2.9 software.

### Fluorimetric Determination of ROS and Superoxide Levels

Reactive oxygen species levels were determined by the membrane-permeant ROS sensitive fluorogenic probe 5,6-carboxy-2′, 7′-dichlorofluoresceindiacetate, DCHF-DA (Molecular Probes, Leiden, The Netherlands). MO3-13 cells were grown to semi-confluence in 24 multiwell plates and incubated for 18 h in medium containing 0.2% FBS before the experiments. Cells were preincubated for 30 min with NAC (200 μM) or SOD1 (400 μg/ml). The cells were washed twice with PBS and incubated in the same buffer with 10 μM DCHF-DA for 10 min. Then, cells were washed three times with PBS containing 10 mM glucose, 1.2 mM MgCl_2_ and 1.2 mM CaCl_2_ and dichlorofluorescein (DCF) fluorescence was measured at different time intervals using the plate reader Fluoroskan Ascent FL fluorometer (Thermo Electron Oy, Vantaa, Finland) and data analyzed by Ascent software.

Superoxide levels were assayed by flow cytometry using two different fluorescent probes, Dihydroethidium (DHE) (Molecular ProbeTM) or the mitochondria-targeted equivalent MitoSOX^TM^Red reagent (Molecular Probe^TM^). MO3-13 cells were grown to semi-confluence in 60-mm culture dishes and incubated for 18 h in medium containing 0.2% FBS.

For the DHE staining the cells were preincubated for 30 min with Cu,Zn superoxide dismutase (SOD1, 400 ng/ml) and stimulated for 30 min with PMA (100 nM). After incubations the cells were washed twice with PBS and incubated with DHE (10 μM) for 30 min at 37°C, protected from light. For the MitoSOX^TM^Red staining the cells were stimulated for 30 min with PMA (100 nM), washed twice with PBS and incubated in the same buffer with 5 μM MitoSOX^TM^Red reagent for 10 min at 37°C, protected from light.

Then, for both assays, the cells were washed twice with PBS, trypsinized and centrifuged at 2000 rpm for 5 min, and then resuspended in PBS. The cells were analyzed using FACSCAN flow cytometer (BD, Heidelberg, Germany) and WINMDI 2.9 software.

### Immunofluorescence Confocal Microscopy

MO3-13 cells were grown on glass coverslip under culture conditions described in the specific experiments. Then, the medium was removed and cells immediately fixed in 3.7% Paraformaldehyde in PBS with 2% Sucrose, pH 7.4, for 5 min at 22°C, and, after two washes in PBS with 2% Sucrose, permeabilized for 10 min at 4°C with 0.01% Saponin (Sigma–Aldrich, from quillaja bark) in PBS for MBP staining; cells were permeabilized for 5 min at 4°C with 0.1%Triton X-100 in 20 mM Hepes, 300 mM Sucrose, 50 mM NaCl, 3 mM MgCl_2_ for Olig-2 staining.

The cells, after blocking with 20% FBS in PBS with 0.01% Saponin for 30 min at 4°C, were labeled with primary rabbit-polyclonal anti human MBP antibody (Millipore Upstate) or after blocking with 20% FBS in PBS for 30 min at 4°C, were labelled with primary rabbit polyclonal anti human Olig-2 antibody (Millipore). The cells were washed and labeled with secondary Cy3-conjugated anti-rabbit IgG (Jackson ImmunoResearch, USA). Controls were incubated with secondary antibodies alone. After treatment with nuclear marker, 4′,6-diamidino-2-phenylindole (DAPI), the coverslips were briefly washed first, in PBS and then in distilled water, and finally mounted on glass slides for microscopy examination. Cells were analyzed with a Zeiss LSM 510 Meta laser scanning confocal microscope.

Afterward, images were analyzed using the ImageJ software: we set the threshold on the maximum fluorescent value of the sample treated only with secondary antibody, and quantified 25 cells for each sample. Regions of interest (ROI) were defined to restrict the analysis to a spatially confined cellular area.

### RNA Interference

Human NOX3, NOX5 and PKCα small interfering RNAs ON-TARGETplus (siRNAs) were obtained from Dharmacon (USA). Transfection was carried out by Neon Transfection System by Life Technology (Invitrogen). The experimental conditions were optimized for MO3-13 cells: voltage 1700, width 20 ms, 1 pulse and transfection efficiency (70 ± 7%) was evaluated using 2 μg of Green Fluorescent Protein (GFP). Percent of GFP positive cells were evaluated after 48 h of transfection by flow cytometry using FACSCAN (BD, Heidelberg, Germany) and WINMDI software.

Cells were dissociated by a brief treatment with trypsin-ethylenediaminetetraacetic acid (EDTA), and counted. siRNAs were introduced into each 1 × 10^6^ dissociated cells in 300 μl volume according to manufacturer’s instructions; electroporated cells were then seeded into culture dishes containing pre-warmed culture media.

NOX3 and NOX5 knockdowns were tested by immunoblot and RT-PCR too. PKCα knockdown was tested by immunoblot for p-PKCα. As controls were used “non-targeting” (NT) scrambled siRNAs. In all experiments siRNAs were used at a final concentration of 100 nM.

### RNA Isolation

Total RNA was extracted using TRIzol reagent according to the protocol provided by the manufacturer (Sigma–Aldrich). Total RNA (1 μg) was reverse transcribed using Transcriptor First Strand cDNA Synthesis Kit (Roche Applied Science) by oligo-dT primers for 30 min at 55°C in a 20 μl reaction volume.

### Qualitative PCR

Qualitative PCR was performed using Hot Master Taq DNA Polymerase (5PRIME) in 20 μl final volume containing 0.2 mM dNTP, 0.2 μM of the specific primers and 100 ng of sample cDNA. The PCR conditions used were 94°C 2 min, (94°C 30 s, 60°C 30 s, 70°C 30 s) and 70°C 5 min in GeneAmp PCR Sysyem 9700 (Applied Biosystem Inc, USA). The reactions were carried out at different cycles (30–35).

### Real-Time PCR

Real-time PCR was performed using LightCycler480 II System (Roche) in 96-well optical reaction plates and in a 20 μl final volume containing 10 μl of LightCycler480 SYBR GREEN I Master (Roche Applied Science), 1 μl of each gene-specific primer and 50 ng of sample cDNA. Gene-specific primers were designed to selectively amplify MBP, Olig-2, NOX1, NOX2, NOX3, NOX4 and NOX5, and relative expression values were normalized using glyceraldehyde 3-phosphate dehydrogenase (GAPDH). The SYBR GREEN fluorescence was measured at each extension step. The Threshold Cycle (Ct) reflects the cycle number at which the fluorescence generated crosses an arbitrary threshold. Melting curve analysis was performed to determine the specificity of the reaction. Real-time PCR was conducted in triplicate for each sample and the mean value was calculated.

Primers used in these studies are the following:

MBP: (F), CTC CAT CGG GCG CTT CTT TG (R), CGG GTG GTG TGA GTC CTT GT
OLIG-2: (F), CCA GAG CCC GAT GAC CTT TTT (R), CAC TGC CTC CTA GCT TGT C
Human NOX1 (F), TTA ACA GCA CGC TGA TCC TG (R), CAC TCC AGT GAG ACC AGC AA
Human cytochrome b-245, beta polypeptide (CYBB, *alias* NOX2): (F), GGA GTT TCA AGA TGC GTG GAA ACT A (R), GCC AGA CTC AGA GTT GGA GAT GCT
Human NOX 3: (F), CCA GGG CAG TAC ATC TTG GT (R), CCG TGT TTC CAG GGA GAG TA
Human NOX4: (F), GCT TAC CTC CGA GGA TCA CA (R), CGG GAG GGT GGG TAT CTA A
Human NOX5: (F), ATC AAG CGG CCC CCT TTT TTT CAC (R), CTC ATT GTC ACA CTC CTC GAC AGC
Human GAPDH: (F), AGG CTG AGA ACG GGA AGC (R), CCA TGG TGG TGA AGA CGC

### Statistical Analysis

Statistical differences were evaluated using a Student’s *t*-test for unpaired samples.

## Results

### Differential Effects of H_2_O_2_ on Viability of MO3-13 Precursor or Differentiated Cells

To evaluate whether ROS influence human OLs differentiation, we analyzed the effects of H_2_O_2_ on the expression of specific OL differentiation markers. To this end, we used human OL MO3-13 cells which express phenotypic markers of primary OPCs. These cells, when cultured in serum-free medium, supplemented with 100 nM of PMA arrest growth and differentiate to mature OLs (McLaurin et al., 1995; Buntinx et al., 2003).

**FIGURE 1:**
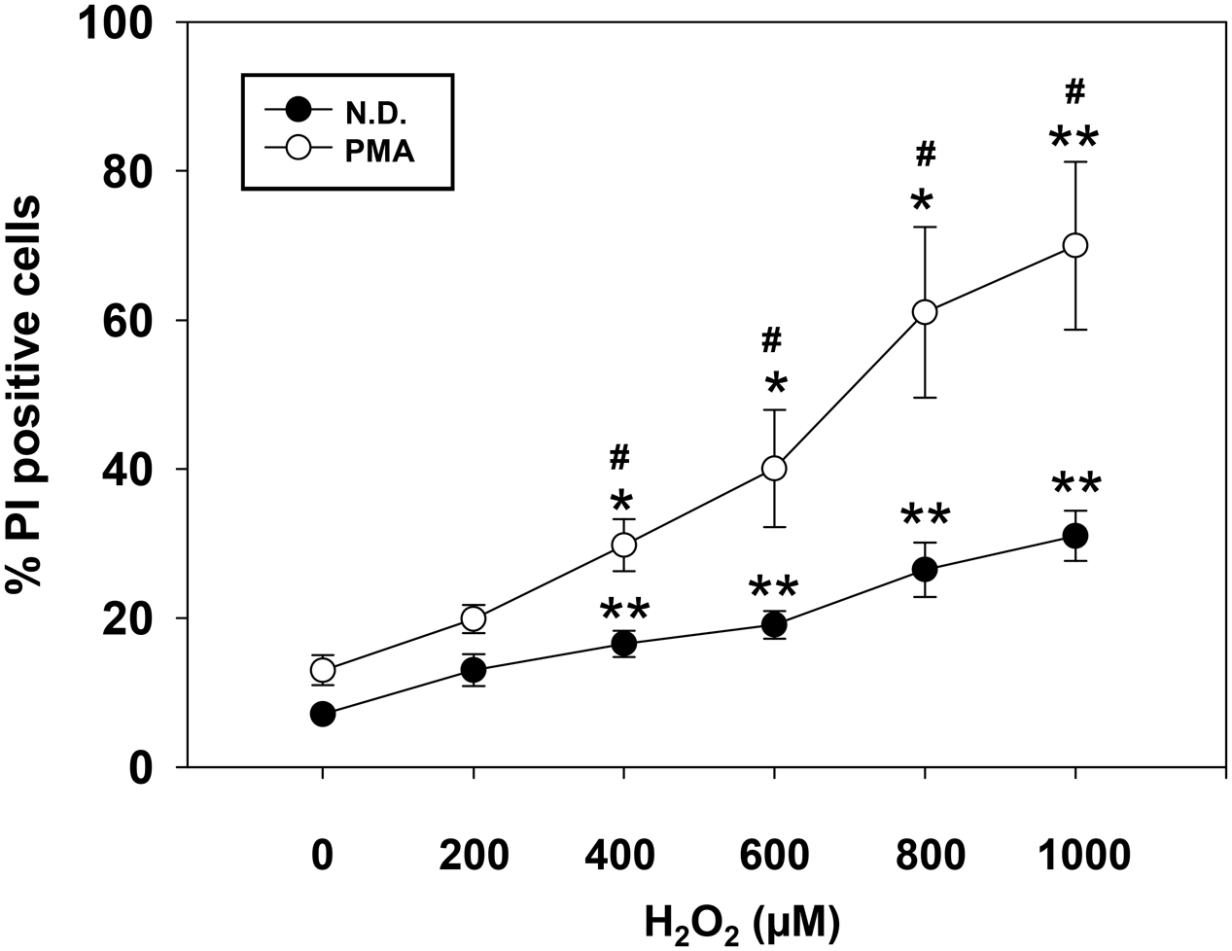
**Dose-dependent effect of H_2_O_2_ on cell viability.** Not differentiated (N.D.), growing in complete medium, and differentiated (PMA) MO3-13 cells were stimulated with increasing doses of H_2_O_2_ and cell viability was evaluated by cytofluorimetry after Propidium Iodide (PI) staining. The MO3-13 cells were differentiated in FBS-free DMEM supplemented with 100 nM of PMA for 4 days. The graphs show the mean ± SEM values relative to control of three independent experiments. ^∗^*p* < 0.05, ^∗∗^*p* < 0.01 vs. Control; ^#^*p* < 0.05 vs. Corresponding dose.

To investigate the impact of H_2_O_2_ on OL differentiation, we first determined the survival of cells exposed to increasing concentration of H_2_O_2_. Cell viability was assessed by flow-cytometric analysis either on precursor or on PMA-differentiated MO3-13 cells in the absence (**Figure 1**) or in the presence of serum (**Supplementary Figure S1**). Differentiated cells were more susceptible to death induced by high oxidative stress in both conditions (with or without serum). On the basis of dose-response curves we conclude that 200 μM of H_2_O_2_, did not alter survival of differentiated or undifferentiated cells. This is the main reason why we used this dose in all the subsequent experiments.

### MO3-13 Cells Exposed to H_2_O_2_ Differentiate

MO3-13 cells express low levels of the OL differentiation markers MBP and Olig-2 (**Supplementary Figure S2**). MBP is one of the major proteins of CNS myelin sheath and constitutes as much as 30% of its protein content (Baumann and Pham-Dinh, 2001), while Olig-2 is a specific OL transcription factor essential for OL lineage commitment both *in vivo* and *in vitro* (Lu et al., 2002; Samanta and Kessler, 2004).

**FIGURE 2:**
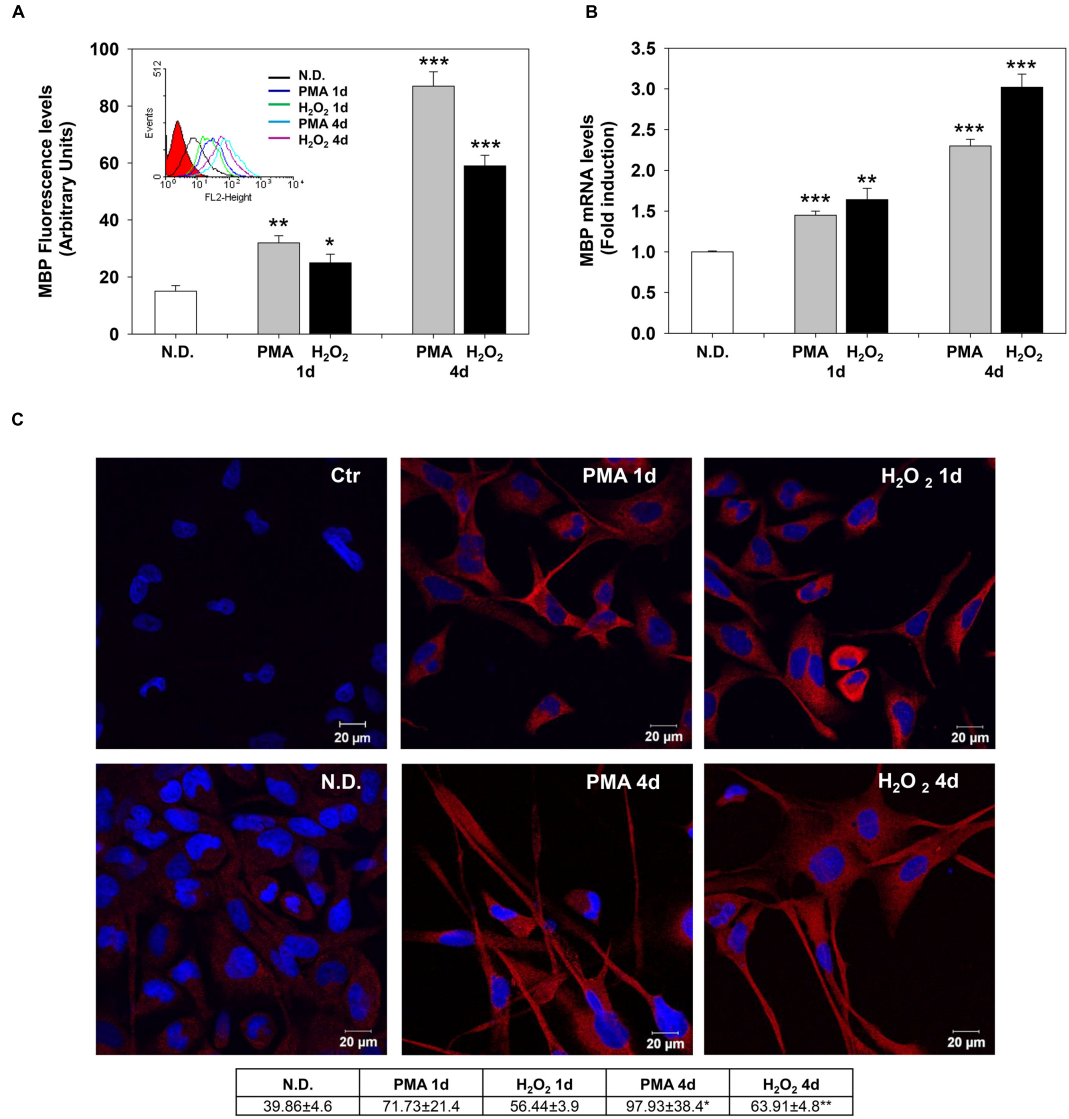
**Phorbol-12-*Myristate*-13-Acetate and H_2_O_2_ induce MBP expression levels.** **(A)** MBP protein levels in MO3-13 cells treated for 1 and 4 days with 100 nM PMA in medium without serum or with 200 μM H_2_O_2_. Immunoreactivity for MBP was evidenced by indirect immunofluorescence and flow cytometric analysis, using primary antibodies against MBP and CY3-conjugated anti rabbit IgG as secondary antibodies. 10,000 cells were counted for each sample. N.D. indicates Not Differentiated cells, growing in complete medium. The graph shows the mean ± SEM values of three independent experiments. The insert shows the histograms of a representative experiment; the red histogram denotes the sample treated with secondary antibodies alone. **(B)** Real-time-PCR analysis of MBP mRNA levels in MO3-13 cells treated for 1 and 4 days with 100 nM PMA in medium without serum or with 200 μM H_2_O_2_. N.D. indicates Not Differentiated cells, growing in complete medium. Expression values were normalized using glucose-6-phosphate-dehydrogenase mRNA (G6PD). The histogram shows means ± SEM values relative to control of three independent experiments. **(C)** Confocal images of MBP immunoreactivity of MO3-13 cells after 1 and 4 days of differentiation with 100nM PMA in medium without serum or with 200 μM H_2_O_2_. Cells were stained with the nuclear dye DAPI (blue) and with primary anti human MBP antibodies and CY3-conjugated anti rabbit IgG as secondary antibodies (red). Control (Ctr) was treated with secondary antibodies and DAPI alone. N.D. indicates Not Differentiated cells, growing in complete medium. The table under the images shows means ± SEM values obtained by the quantitative analysis of 25 cells for each sample. ^∗^*p* < 0.05, ^∗∗^*p* < 0.01, ^∗∗∗^*p* < 0.001 vs. N.D.

The effects of PMA and H_2_O_2_ on these two differentiation markers were evaluated after 1 and 4 days of treatment. Indirect immunofluorescence and flow cytometric analysis showed that MBP levels progressively increase after 1 and 4 days of treatment (**Figure 2A**). Similarly, PMA or H_2_O_2_ progressively increased mRNA levels of MBP after 1 and 4 days of treatment (**Figure 2B**).

**FIGURE 3:**
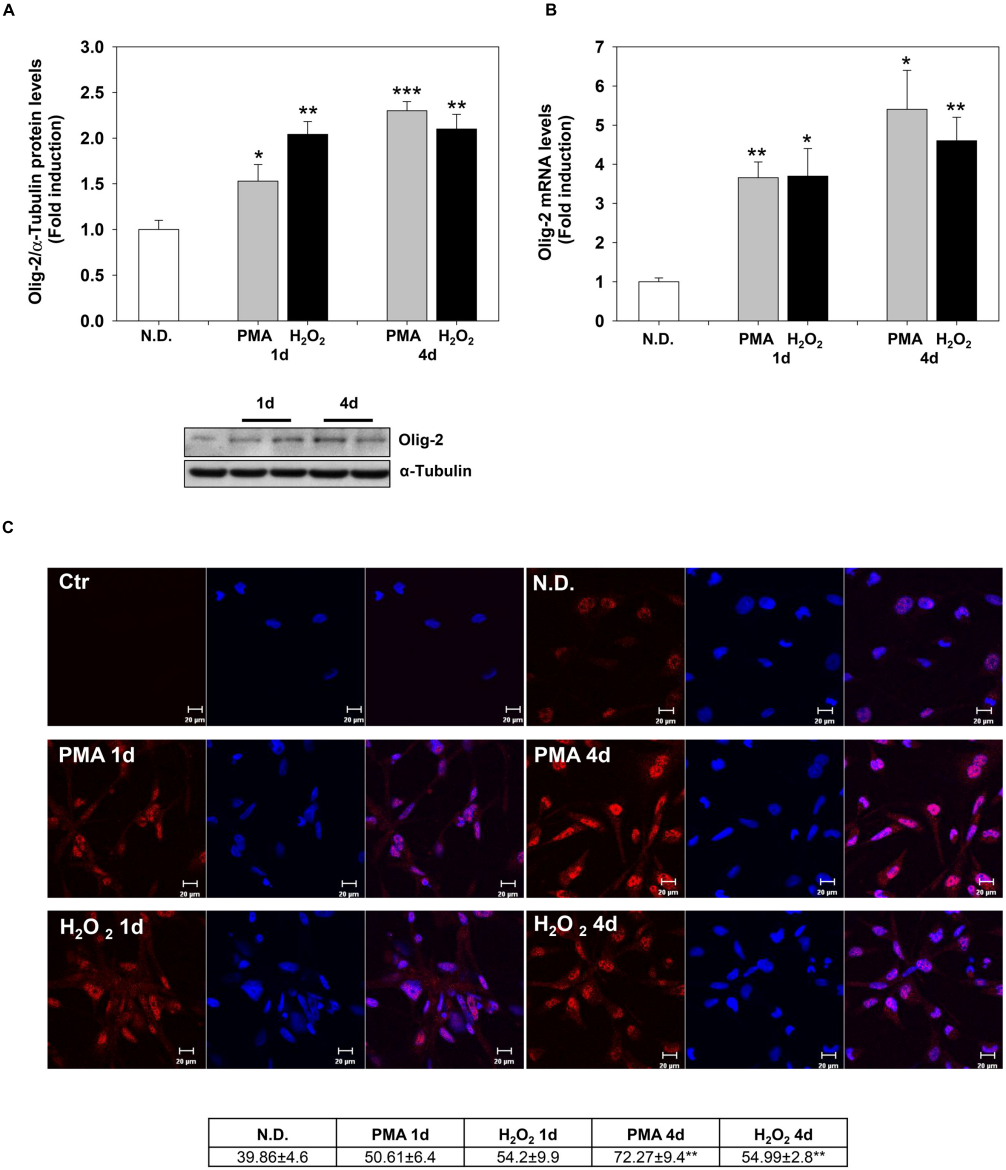
**Phorbol-12-*Myristate*-13-Acetate and H_2_O_2_ induce Olig-2 expression levels.** **(A)** Western blotting analysis of Olig-2 levels in MO3-13 cells treated for 1 and 4 days with 100 nM PMA in medium without serum or with 200 μM H_2_O_2_. N.D. indicates Not Differentiated cells, growing in complete medium. The histogram shows the values (means ± SEM) relative to control obtained by densitometric analysis of Olig-2 normalized to α-Tubulin of three independent experiments. **(B)** Real-time-PCR analysis of Olig-*2* m-RNA levels in MO3-13 cells treated for 1 and 4 days with 100 nM PMA in medium without serum or with 200 μM H_2_O_2_. N.D. indicates Not Differentiated cells, growing in complete medium. Expression values were normalized using glucose-6-phosphate-dehydrogenase mRNA (G6PD). The histogram shows means ± SEM values relative to control of three independent experiments. **(C)** Confocal images of Olig-2 immunoreactivity of MO3-13 cells after 1 and 4 days of differentiation with 100 nM PMA in medium without serum or with 200 μM H_2_O_2_. Cells were stained with the nuclear dye DAPI (blue) and with primary anti human Olig-2 antibodies followed by CY3-conjugated anti rabbit IgG as secondary antibodies. Control (Ctr) was treated with secondary antibodies and DAPI alone. N.D. indicates Not Differentiated cells, growing in complete medium. For each image are shown three panels: on the left Olig-2 (red); on the center nuclei (blue); on the right the merged image. The table under the images shows means ± SEM values obtained by the quantitative analysis of 25 cells for each sample. ^∗^*p* < 0.05, ^∗∗^*p* < 0.01, ^∗∗∗^*p* < 0.001 vs. N.D.

Confocal microscopy confirmed the increase of MBP levels after PMA or H_2_O_2_ treatment (**Figure 2C**). The distribution of the protein in the cell processes and membrane patches confirms the differentiation in mature OLs (Barbarese et al., 1988). Also, Olig-2 protein (**Figure 3A**) and mRNA (**Figure 3B**) levels significantly increased following 1 or 4 days of treatment with PMA or H_2_O_2_. Confocal microscopy analysis confirmed the increase of Olig-2 protein after PMA or H_2_O_2_ treatments (**Figure 3C**).

Increased expression of MBP and Olig-2 is associated with higher phosphorylation levels of extracellular signal regulated kinase 1-2 (ERK1-2) and of cyclic AMP responsive element binding protein 1 (CREB), which are selectively activated by neurotransmitters or growth factors at specific stages of OL development (Sato-Bigbee et al., 1999; Johnson et al., 2000; Fyffe-Maricich et al., 2011). ERK1-2 and CREB control OPCs differentiation at early (Syed et al., 2013) and late stages, promoting myelin growth in fully differentiated myelinating OLs (Afshari et al., 2001; Ishii et al., 2013).

**FIGURE 4:**
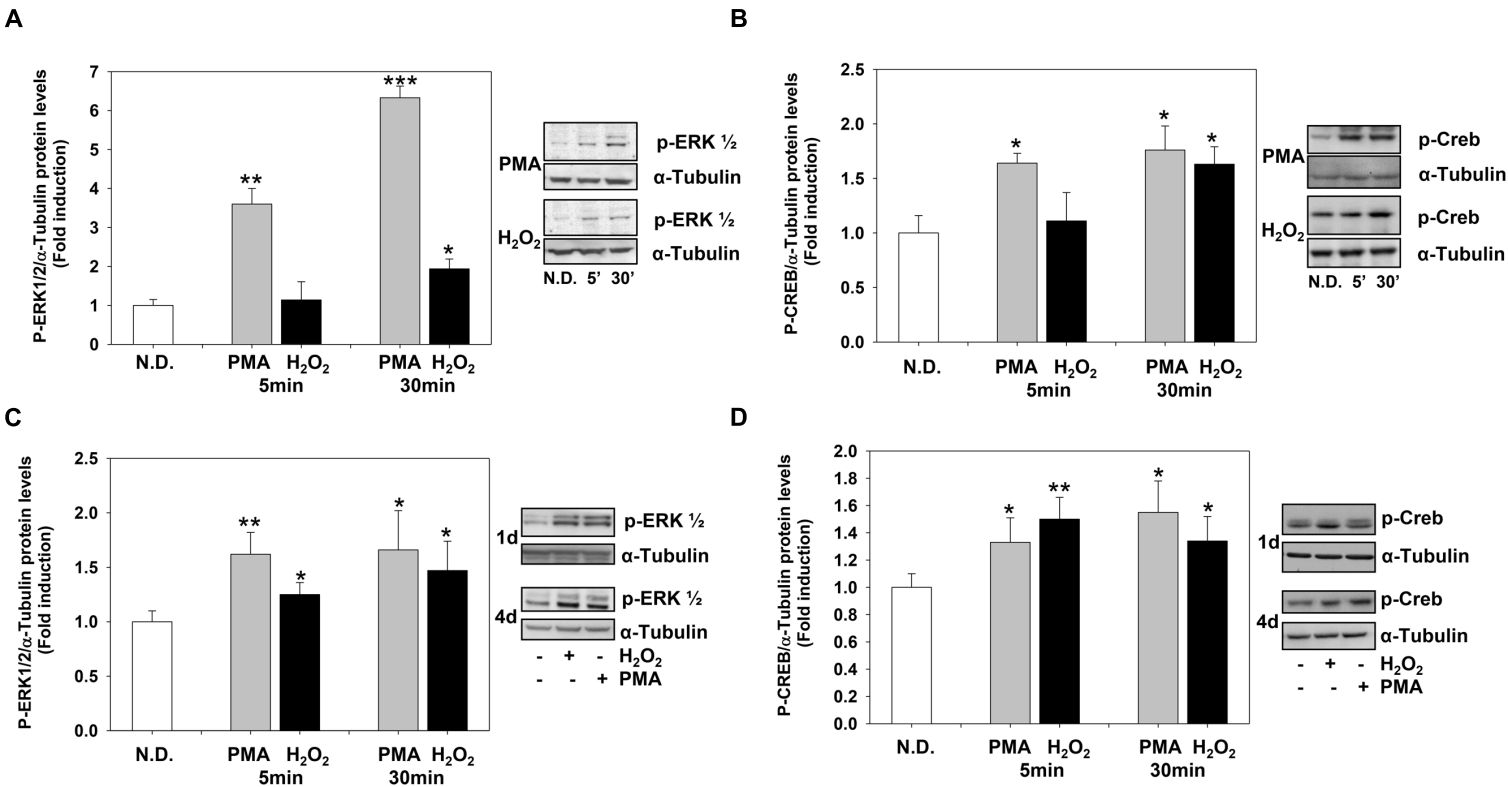
**Phorbol-12-*Myristate*-13-Acetate and H_2_O_2_ induce ERK1-2 and CREB phosphorylation levels.** **(A,B)** Western blotting analysis of p-ERK1-2 **(A)** and p-CREB **(B)** levels in MO3-13 cells treated for 5 or 30 min with 100 nM PMA in medium without serum or with 200 μM H_2_O_2_. **(C,D)** Western blotting analysis of p-ERK1-2 **(C)** and p-CREB **(D)** levels in MO3-13 cells treated for 1 or 4 days with 100 nM PMA in medium without serum or with 200 μM H_2_O_2_. N.D. indicates Not Differentiated cells, growing in complete medium. The histograms show the values (means ± SEM) relative to control (N.D.) obtained by densitometric analysis of protein bands normalized to α-Tubulin of three independent experiments. ^∗^*p* < 0.05, ^∗∗^*p* < 0.01, ^∗∗∗^*p* < 0.001 vs. N.D.

The levels of P-ERK1-2 and P-CREB increased rapidly after PMA or H_2_O_2_ stimulation. PMA and H_2_O_2_ significantly induced P-ERK1-2 and P-CREB levels as soon as 5 and 30 min, respectively (**Figures 4A,B**) and the levels of both phosphoproteins remained high during the 4 days of treatment (**Figures 4C,D**).

### PKC Signaling Mediate H_2_O_2_-Induced OPC Differentiation

**FIGURE 5:**
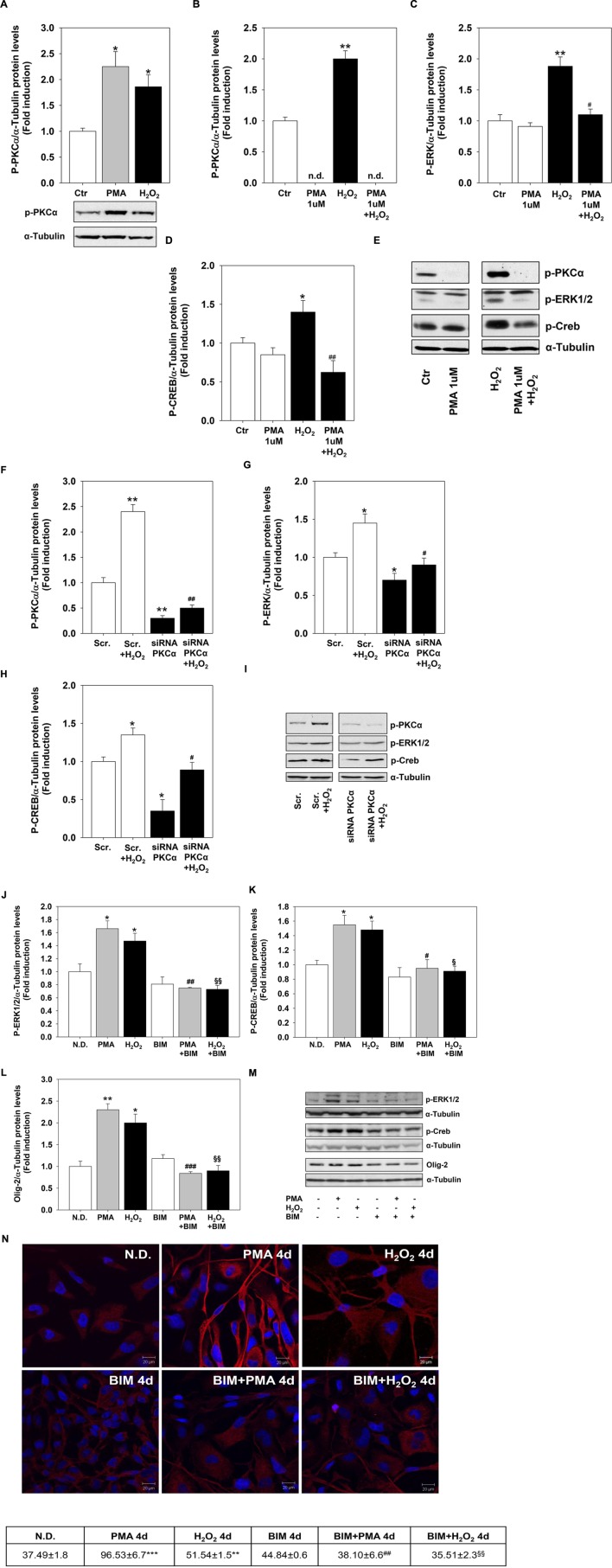
**Protein kinase C mediates H_2_O_2_ effects.** **(A)** Western blotting analysis of P-PKCα expression levels in MO3-13 cells incubated for 18 h in medium containing 0.2% FBS and then stimulated with H_2_O_2_ (200 μM) or PMA (100 nM) for 30 min. The histogram shows the values (means ± SEM) relative to control obtained by densitometric analysis of protein bands normalized to α-Tubulin of three independent experiments. Under the histogram is shown a representative experiment.^∗^*p* < 0.05 vs. control (Ctr). **(B–E)** PKC depletion was obtained by treatment of MO3-13 cells for 24 h with 1 μM PMA in medium containing 0.2% FBS. Then cells were stimulated for 30 min with H_2_O_2_ (200 μM) before Western blotting analysis of p-PKCα **(B)**, P-ERK1-2 **(C)** and P-CREB **(D)** expression levels. n.d., not detectable. The histograms show the values (means ± SEM) relative to control obtained by densitometric analysis of protein bands normalized to α-Tubulin of three independent experiments. **(E)** A representative experiment is shown. ^∗^*p* < 0.05, ^∗∗^*p* < 0.01 vs. control (Ctr); ^#^*p* < 0.05, ^##^*p* < 0.01 vs. H_2_O_2_. **(F–I)** MO3-13 cells were transfected by electroporation with siRNA to PKCα or control, scrambled siRNA (Scrambled) as described in Materials and Methods. 24 h after transfection, cells were stimulated with H_2_O_2_ (200 μM) for 30 min in medium containing 0.2% FBS and total proteins were extracted and subjected to immunoblot analysis of p-PKCα **(F)**, P-ERK1-2 **(G)** and P-CREB **(H)** expression levels. The histograms show the values (means ± SEM) relative to control obtained by densitometric analysis of protein bands normalized to α-Tubulin of three independent experiments. **(I)** A representative experiment is shown. ^∗^*p* < 0.05, ^∗∗^*p* < 0.01 vs. Scrambled (Scr.); ^#^*p* < 0.05, ^##^p < 0.001 vs. Scrambled (Scr.) + H_2_O_2_. **(J–M)** Western blotting analysis of p-ERK1-2 **(J)** p-CREB **(K)**, and Olig-2 **(L)** expression levels in MO3-13 cells stimulated with H_2_O_2_ (200 μM) or PMA (100 nM) for 4 days in the presence or absence of BIM (100 μM). N.D. indicates Not Differentiated cells, growing in complete medium. The histograms show the values (means ± SEM) relative to control obtained by densitometric analysis of protein bands normalized to α-Tubulin of three independent experiments. **(M)** A representative experiment is shown. **(N)** Confocal images of MBP immunoreactivity of MO3-13 cells after 4 days of treatment with 100 nM PMA in medium without serum or with 200 μM H_2_O_2_ in the presence or absence of BIM (100 μM). Cells were stained with nuclear dye DAPI and with primary anti human MBP antibodies and CY3-conjugated anti rabbit IgG as secondary antibodies. N.D. indicates Not Differentiated cells, growing in complete medium. The table under the images shows means ± SEM values obtained by the quantitative analysis of 25 cells for each sample. ^∗^*p* < 0.05, ^∗∗^*p* < 0.01, ^∗∗∗^*p* < 0.001 vs. N.D.; ^#^*p* < 0.05, ^##^*p* < 0.01, ^###^*p* < 0.001 vs. PMA; ^§^ *p* < 0.05, ^§§^ *p* < 0.01 vs. H_2_O_2_.

To evaluate whether PMA and H_2_O_2_ effects on MO3-13 precursor cells differentiation are linked and converge on the PKC pathway, we first assessed the activation of PKC by H_2_O_2_. Both treatments increased the phosphorylation levels of the α subunit of PKC (**Figure 5A**). Then, we depleted the cells of PKC by long-term treatment (24 h) with 1 μM PMA in medium containing 0.2% FBS before stimulating them for 30 min with H_2_O_2_ 200 μM. This treatment leads to a strong down-regulation of P-PKCα levels that become undetectable by immunoblot (**Figure 5B**). PKC depletion inhibits the induction of CREB and ERK1-2 phosphorylation levels by H_2_O_2_ (**Figures 5C–E**). Similar results were obtained by knock-down experiments by using specific PKCα siRNA (**Figures 5F–I**).

To further evaluate whether PCK signaling mediates H_2_O_2_ effects on MO3-13 precursor cells differentiation we used the PKC α-β-γ inhibitor BIM. **Figures 5J–M** shows that pretreatment of the cells with, BIM completely reversed the induction by PMA or H_2_O_2_ of P-ERK1-2, P-CREB and Olig-2. The PKC inhibitor also reversed long term effects of PMA and H_2_O_2_ (4 days) on MBP protein levels as shown by confocal microscopy analysis (**Figure 5N**).

These data link H_2_O_2_ and PKC signals and suggest that PKC is upstream and downstream H_2_O_2_ because the PKC depletion or inhibition prevents H_2_O_2_ induction of differentiation.

### ROS Derived from NOX3 and NOX5 Mediate PMA-Induced MO3-13 Cells Differentiation

**FIGURE 6:**
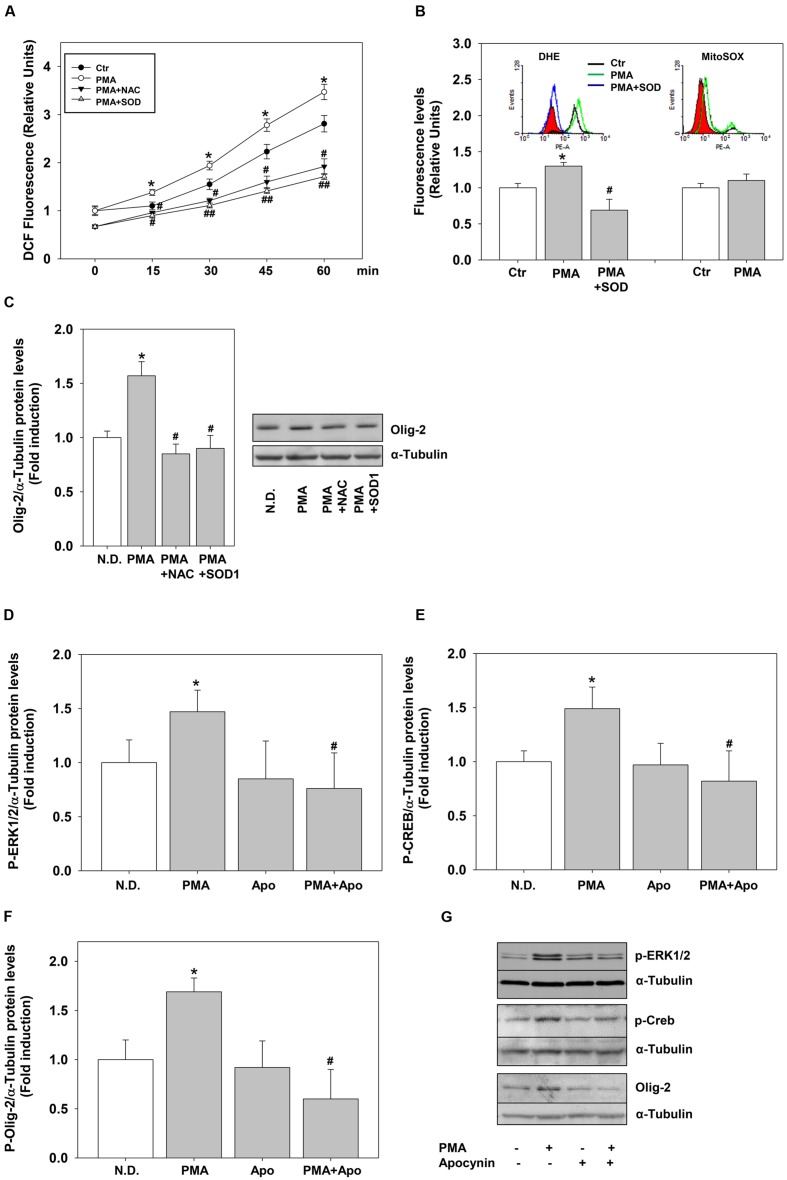
**Phorbol-12-*Myristate*-13-Acetate pro-differentiative effects rely on NOX dependent ROS.** **(A)** MO3-13 cells were grown in complete medium, preincubated with antioxidants (200 μM NAC or 400 μg/ml SOD1), incubated with 10 μM of the ROS sensitive probe DCHF-DA and then stimulated with PMA 100 nM in absence of serum. ROS levels were measured by fluorometric analysis at various times.The graphs show the mean ±SEM values relative to control of three independent experiments. ^∗^*p* < 0.05 vs. corresponding time points of not stimulated samples (Ctr) ^#^*p* < 0.05, ^##^*p* < 001 vs. corresponding time points of PMA stimulated samples. **(B)** MO3-13 cells were incubated for 18 h in medium containing 0.2% FBS. For the DHE staining the cells were preincubated for 30 min with SOD1 (400 ng/ml) and stimulated for 30 min with PMA (100 nM). For MitoSOX staining cells were incubated for 30 min with PMA (100 nM). Superoxide levels were measured by flow cytometry. The graphs show the mean ±SEM values relative to control (Ctr) of three independent experiments. The inserts show the hystograms of representative experiments; the red histogram denotes the sample treated with secondary antibodies alone. ^∗^*p* < 0.05 vs. control; ^#^*p* < 0.05 vs. PMA. **(C)** MO3-13 cells were grown in complete medium, pre-incubated in the absence and presence of 200 μM NAC or 400 μg/ml SOD1 for 30 min and stimulated for 4 days with 100 nM PMA in the absence of serum before being collected and analyzed by Western Blotting. N.D. indicates Not Differentiated cells, growing in complete medium. The histogram shows the values (means ± SEM) relative to control obtained by densitometric analysis of Olig-2 normalized to α-tubulin compared to undifferentiated control of three independent experiments. ^∗^*p* < 0.02 vs. N.D.; ^#^*p* < 0.05 vs. PMA **(D–G)** Western blotting analysis of p-ERK1-2 **(D)**, p-CREB **(E)** and Olig-2 **(F)** expression levels in MO3-13 cells stimulated with PMA (100 nM) for 4 days in serum-free medium in the presence or absence of Apocynin (50 μM). N.D. indicates Not Differentiated cells, growing in complete medium. The histograms shows the values (means ± SEM) relative to control obtained by densitometric analysis of protein bands normalized to α-Tubulin compared to undifferentiated control of three independent experiments. ^∗^*p* < 0.05 vs. N.D.; ^#^*p* < 0.05 vs. PMA. **(G)** A representative experiment is shown.

To define precisely the link between H_2_O_2_ and PKC we measured the effects of PMA on ROS levels. PMA stimulation significantly and rapidly (15 min) induced ROS levels, measured as DCF fluorescence (**Figure 6A**). ROS levels remained higher compared to the controls for 4 days of continuous treatment (data not shown). To evaluate the nature of DCF signal we used two different superoxide fluorescent probes, DHE or the mitochondria-targeted equivalent MitoSOX–Red. Flow cytometry experiments shows that PMA increases superoxide levels (DHE staining) without affecting mitochondrial superoxide production (Mito-SOX-Red staining) (**Figure 6B**).

To demonstrate a direct link between PMA-induced ROS and cell differentiation, we evaluated the effects of the generic ROS scavenger NAC and of the superoxide scavenger enzyme superoxide dismutase1 (SOD1). Externally added SOD1 is internalized by endocytosis exerting intracellular antioxidant activity (Dini and Rotilio, 1989; Mondola et al., 2002). The antioxidants were able not only to reduce PMA-dependent ROS production but also PMA-induction of Olig-2 (**Figures 6A–C**).

**FIGURE 7:**
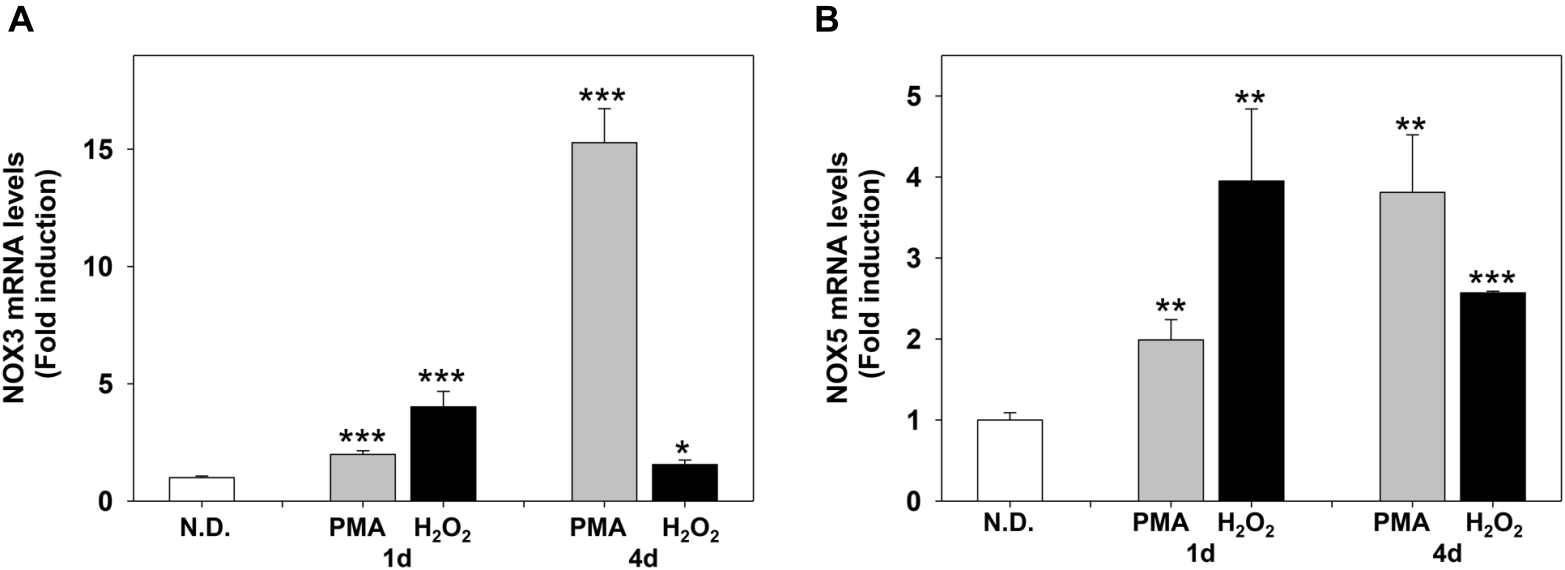
**Phorbol-12-*Myristate*-13-Acetate and H_2_O_2_ induce NOX3 and NOX5 m-RNA levels in MO3-13 cells.** Real-time-PCR analysis of NOX3 **(A)** and NOX5 **(B)** mRNA levels in MO3-13 cells treated for 1 and 4 days with 100 nM PMA in medium without serum or with 200 μM H_2_O_2_. N.D. indicates Not Differentiated cells, growing in complete medium. Expression values were normalized using glucose-6-phosphate-dehydrogenase mRNA (G6PD). The histograms show means ± SEM values relative to control in eight independent experiments. ^∗^*p* < 0.05, ^∗∗^*p* < 0.01, ^∗∗∗^*p* < 0.001 vs. N.D.

The main sources of ROS are NOX enzymes and the data shown above suggest that PMA and/or H_2_O_2_ may induce these enzymes. We measured the expression of many NOX-DUOX enzymes in MO3-13 cells by semi-quantitative and real-time PCR. Besides the dual oxidases DUOX1 and 2 (Damiano et al., 2012), we found that MO3-13 cells express NOX3 and NOX5 (**Supplementary Figure S3**; **Figure 7**). Therefore, to test whether these enzymes were mediators of PMA effects on MO3-13 precursor cells differentiation, we exposed the cells stimulated by PMA to apocynin. Apocynin is an inhibitor of NOX enzymes; specifically it inhibits the interaction of the cytosolic subunit p47phox with the catalytic membrane subunit gp91phox of NOXs and therefore apocynin inhibits NOX1, NOX2, and NOX3, enzymes that are dependent for their activity on membrane translocation of cytosolic subunits. **Figures 6D–G** shows that in the presence of apocynin, PMA failed to induce P-ERK1-2, P-CREB and Olig-2 levels (after 4 days of treatment), suggesting an involvement of NOXs-derived ROS in PMA-induced differentiation.

We next evaluated the effects of PMA and H_2_O_2_ on NOX3 and NOX5 expression. **Figures 7A,B** show that PMA and H_2_O_2_ induce NOX3 and NOX5 mRNA levels in MO3-13 cells. The timing of induction was slightly different: H_2_O_2_ induced NOX3 and NOX5 mRNA in 1 day, while PMA progressively increased NOX3 and NOX5 in 4 days of treatment.

**FIGURE 8:**
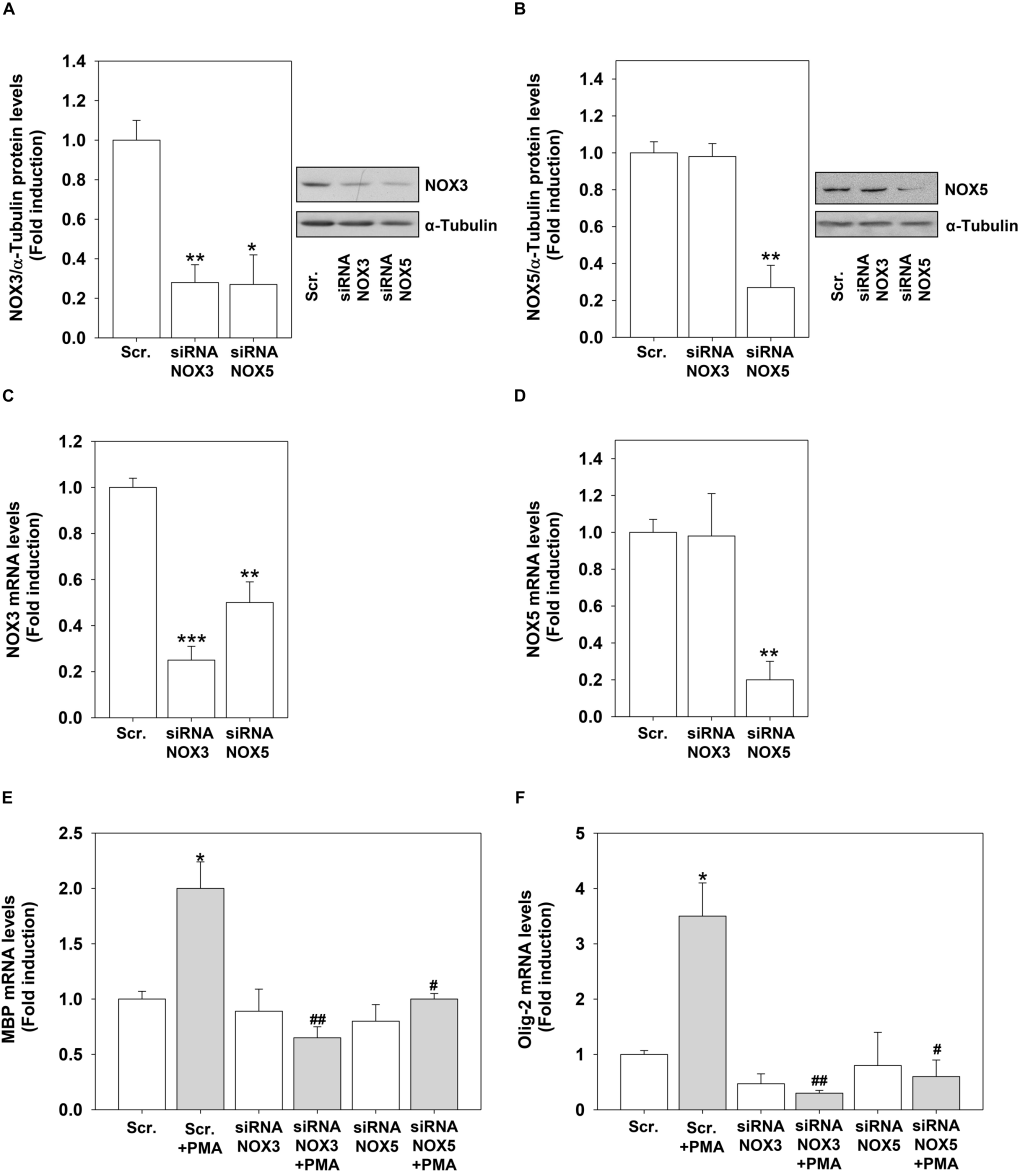
**NOX3 or NOX5 silencing inhibits PMA-induced differentiation.** MO3-13 cells were transfected by electroporation with siRNA to NOX3 (siRNA NOX3) or NOX5 (siRNA NOX5) or control, scrambled siRNA (Scrambled) as described in Section “Materials and Methods.” Twenty four hours after transfection, total proteins were extracted and subjected to immunoblot analysis of NOX3 **(A)** and NOX5 **(B)**. The histograms show the mean ±SEM values relative to control scrambled obtained by densitometric analysis of proteins bands normalized for α-Tubulin of three independent experiments. A representative experiments is shown on the left of each histogram. ^∗^*p* < 0.05 vs. Scrambled. **(C,D)** mRNA from cells treated as in **(A)** was extracted and NOX3 **(C)** or NOX5 **(D)** mRNA levels were analyzed by Real-time-PCR as described in Section “Materials and Methods.” The graphs show the mean ± SEM values relative to scrambled control of three independent experiments. **(E,F)** mRNA from cells treated as in **(A)** was extracted and MBP **(E)** or Olig-2 **(F)** mRNA levels were analyzed by Real-time-PCR as described in Section “Materials and Methods.” The graphs show the mean ±/ SEM values relative to scrambled control of three independent experiments. ^∗^*p* < 0.05, ^∗∗^*p* < 0.01, ^∗∗∗^*p* < 0.001 vs. Scrambled; ^#^*p* < 0.05, ^##^*p* < 0.01, vs. Scrambled + PMA.

To demonstrate the direct link between the increased expression of NOX3 and NOX5 by PMA and H_2_O_2_ and their role in OL differentiation, we performed knock-down experiments by using specific NOX3 and NOX5 siRNA. We were able to significantly reduced NOX3 and NOX5 protein and mRNA levels in OLs (**Figures 8A–D**). Unexpectedly, we found that NOX5 silencing down-regulated basal protein and mRNA levels of NOX3 (**Figures 8A,C**). NOX3 silencing did not modify basal NOX5 expression levels (**Figures 8B,D**). These data indicate that NOX5 regulates the expression of NOX3. Inhibition of both genes prevented PMA effects on MBP and Olig-2 mRNA levels, demonstrating that NOX3 and NOX5 are essential for OL differentiation (**Figures 8E,F**).

## Discussion

We report here that ROS produced by the NOX isoforms NOX3 and NOX5, induce OL differentiation. As cell model, we used the human OL precursor cells MO3-13 that, following PMA treatment in the absence of serum, assume the morphological and biochemical features of mature OLs. To monitor the progress of differentiation, we measured the expression of OLs differentiation markers, MBP and Olig-2 and the phosphorylation of ERK1-2 and CREB.

Reactive oxygen species can induce cellular and DNA damage or differentiation, depending on levels and the source of production. It is known that in rat glial precursor cells the redox state is a central modulator of OLs differentiation (Smith et al., 2000). Our data indicate that ROS produced by NOX3 and NOX5 induce differentiation of OL. However, in MO3-13 cells H_2_O_2_ is bi-functional: at low doses promotes differentiation, at higher doses induces cell death. It is worth noting that differentiated cells become more sensitive to death induced by H_2_O_2_ compared to MO3-13 precursor cells, while proliferating oligodendroglia like (OLN 93) cells are more resistant to the apoptotic effect of H_2_O_2_ compared to differentiated cells (Kameshwar-Rao et al., 1999). The enhanced sensitivity to oxidative stress of differentiated cells may reflect the amplification of a NOX-dependent ROS release leading to an unbalance of the redox state and cell death. In agreement with our findings, NOX inhibitors reduce H_2_O_2_-induced cell death of differentiated neuronal cells (Bertoni et al., 2011).

This is the first report showing the expression of NOX isoforms (3 and 5) in human OLs. The Ca^2+^-dependent NOX5, is expressed in many tissues (Bánfi et al., 2001; Bedard and Krause, 2007) including cells of the cardiovascular system (Bedard et al., 2012). However, NOX5 is absent in rodents and this may explain the lack of data on its expression and function in OLs, because many studies on neuro-glial specification and differentiation were performed with these models. As to NOX3, this enzyme is highly expressed in vestibular and cochlear sensory epithelia and in the spiral ganglion in the inner ear (Bánfi et al., 2004) and its activity has been linked to deafness and to the ototoxicity of drugs and toxins (Mukherjea et al., 2006). Data available on NOX3 in the nervous system are limited, except a report on its increased expression in Alzheimer brain (de la Monte and Wands, 2006).

Our data show that differentiation of OL requires NOX3 and NOX5. PKC seems to be the central inducer of differentiation. PKC is upstream of ROS, because it phosphorylates the cytosolic subunit p47phox stimulating its membrane localization and enzymatic activation (Fontayne et al., 2002). But PKC is also downstream of ROS as shown by PKC activation by H_2_O_2_. The output of ROS-PKC-ROS cascade is the phosphorylation of ERK1-2 and CREB, which can be sustained for 1–4 days. ROS inhibition of tyrosine phosphatases acting on MEK may contribute to the long lasting ERK1-2 and CREB1 phosphorylation (Jiang et al., 2011). Moreover, NOX3 and NOX5 are linked, since depletion of NOX5 reduces the expression of NOX3. We have reported a similar crosstalk between NOXs in another cell system (Damiano et al., 2012). This is not surprising due to the modulation of NOX3 and NOX5 mRNA levels by ROS. In particular, NOX5-derived ROS modulate basal NOX3 expression as shown by the down regulation of NOX3 protein and mRNA levels by NOX5 silencing. On the contrary, even if NOX3 or NOX5 constitutively produces ROS (Ueno et al., 2005; Wang et al., 2014), NOX3 silencing does not affect basal NOX5 expression. Both NOX3 and NOX5 are localized in the plasma-membrane (Brown and Griendling, 2009), and the activity of NOX5 is calcium dependent. We hypothesize that calcium modulation of NOX5 cooperates with ROS in the induction of NOX3 and probably of other genes involved in OL differentiation. PKC is activated by calcium (Cullen, 2003) and apocynin inhibits NMDA-induced OLs differentiation (Cavaliere et al., 2013) or differentiation of MO3-13 precursor cells. Collectively, these data suggest that ROS and calcium are essential for OL differentiation.

**FIGURE 9:**
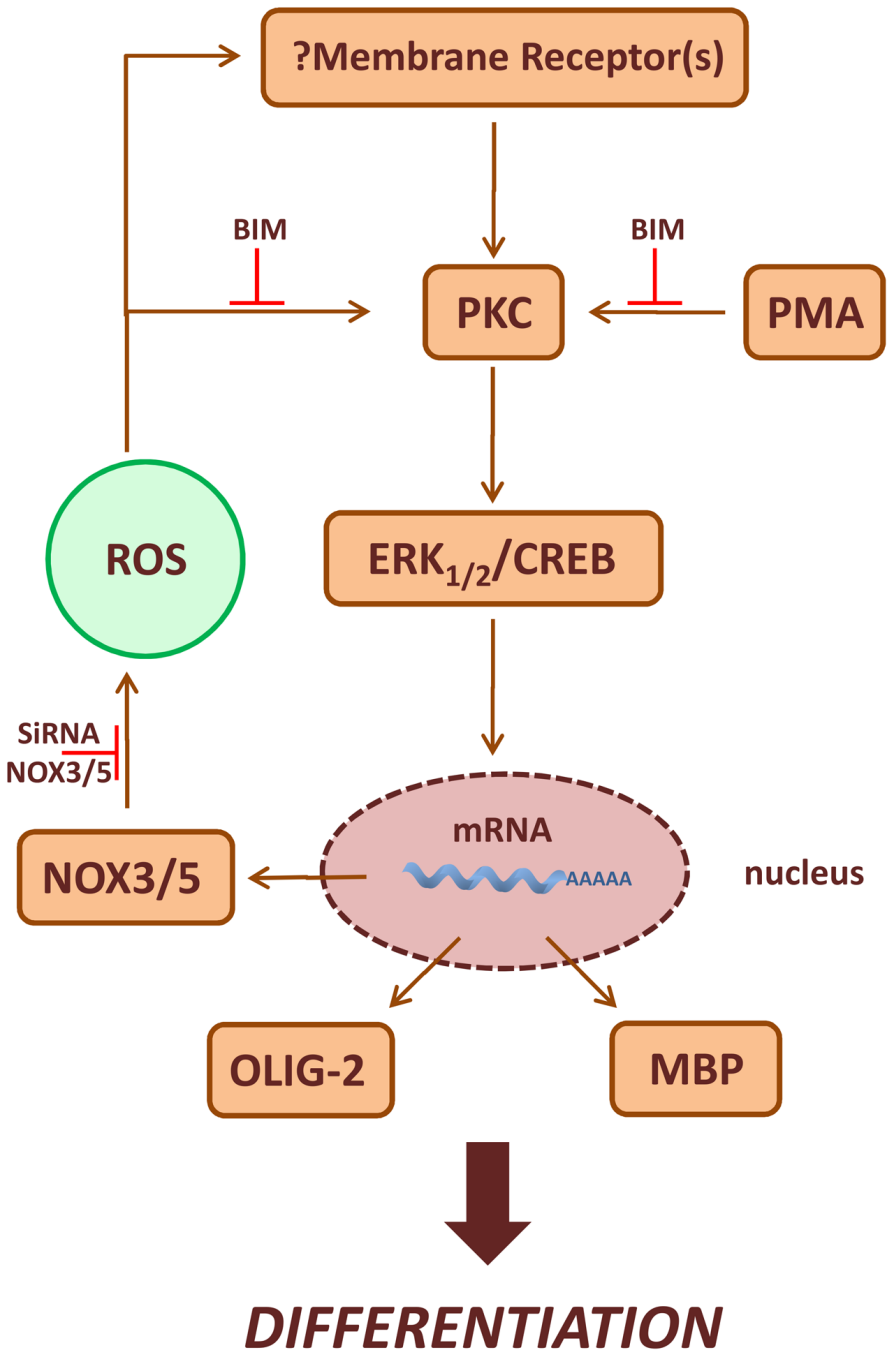
**Scheme of PKC/ROS signaling pathways involved in OLs differentiation.** ROS produced by NOX3/5 amplify PMA activated PKC, ERK1-2 and CREB signaling increasing the expression of the differentiation markers MBP and Olig-2. In the figure is also depicted the possible role of NOX3/5 derived ROS in the stabilization of membrane receptor(s) leading to OLs differentiation.

Many membrane receptors activate NOXs, and ROS produced following ligand-receptor interaction, function as second messenger molecules mediating several cellular events (Serù et al., 2004; Svegliati et al., 2005; Baroni et al., 2006; Gabrielli et al., 2008; Damiano et al., 2012, 2015). P-ERK1-2 and P-CREB levels increase after acute treatment with PMA or H_2_O_2_ in MO3-13 cells and the effects of PMA preceded that of H_2_O_2_. Therefore, we propose that ROS produced by NOX enzymes stabilize membrane receptors involved in OLs differentiation, thus amplifying downstream signals relying on PKC, ERK1-2 and CREB (**Figure 9**). At the moment we do not know which receptor is involved *in vivo* in the redox-dependent signaling leading to OL differentiation and therefore this point needs further investigation. ERK1-2 signaling and CREB phosphorylation seem to have a role in the stimulation of MBP expression and represent signals triggering the final steps of OL maturation (Sato-Bigbee et al., 1999). Accordingly, we found that sustained treatment of MO3-13 cells with H_2_O_2_ exerts long term effects on P-ERK1-2 and P-CREB, demonstrating a role of ROS signaling at early and late steps of OLs differentiation.

A key feature in the development of demyelinating MS lesions is the loss of OLs (Noseworthy et al., 2000). OLs and OPC are highly sensitive to oxidative stress for the low levels of antioxidant defense systems and high intracellular iron content (Thorburne and Juurlink, 1996). It is evident that fine tuning of the type and the levels of ROS generated by NOXs may have profound effects on OPC differentiation and survival.

Traditional disease-modifying therapies for MS based on immunomodulatory and immunosuppressive drugs are mainly aimed at reducing the relapse rate and the formation of new lesions in the relapsing-remitting multiple sclerosis (RRMS) patients, but they are relatively ineffective in preventing the progression of disability. Recently, major attention has been paid to regenerative treatments able to prevent neurodegeneration and favoring remyelination (Zhang et al., 2013). Our data linking OL differentiation with NOX3 and NOX5 expression and activity, suggest that NOX enzymes can represent potential targets of new regenerative therapies ending and reversing the progression of the lesions in MS.

## Author Contributions

MS supervised the entire project, designed research, and wrote the paper. RA conceived and designed the experiments, performed research, interpreted and analyzed data, supervised all the experimental procedure. SD conceived and designed the experiments, performed research interpreted and analyzed data. AM performed research and analyzed data. PM critically revised the manuscript. RP Analyzed data and critically revised the manuscript. EA analyzed data and critically revised the manuscript

## Conflict of Interest Statement

The authors declare that the research was conducted in the absence of any commercial or financial relationships that could be construed as a potential conflict of interest.

## References

[B1] Afshari F. S. Chu A. K. Sato-Bigbee C. (2001). Effect of cyclic AMP on the expression of myelin basic protein species and myelin proteolipid protein in committed oligodendrocytes: differential involvement of the transcription factor CREB. *J. Neurosci. Res.* 66 37–45. 10.1002/jnr.1195 11599000

[B2] Bánfi B. Malgrange B. Knisz J. Steger K. Dubois-Dauphin M. Krause K. H. (2004). NOX3, a superoxide-generating NADPH oxidase of the inner ear. *J. Biol. Chem.* 279 46065–46072. 10.1074/jbc.M403046200 15326186

[B3] Bánfi B. Molnár G. Maturana A. Steger K. Hegedûs B. Demaurex N. (2001). A Ca(2+)-activated NADPH oxidase in testis, spleen, and lymph nodes. *J. Biol. Chem.* 276 37594–37601. 10.1074/jbc.M103034200 11483596

[B4] Barbarese E. Barry C. Chou C. H. Goldstein D. J. Nakos G. A. Hyde-DeRuyscher R. (1988). Expression and localization of myelin basic protein in oligodendrocytes and transfected fibroblasts. *J. Neurochem.* 51 1737–1745. 10.1002/jnr.22570 2460587

[B5] Baroni S. S. Santillo M. Bevilacqua F. Luchetti M. Spadoni T. Mancini M. (2006). Stimulatory autoantibodies to the PDGF receptor in systemic sclerosis. *New Eng. J. Med.* 354 2667–2676. 10.1056/NEJMoa052955 16790699

[B6] Baumann N. Pham-Dinh D. (2001). Biology of oligodendrocyte and myelin in the mammalian central nervous system. *Physiol. Rev.* 81 871–927. 11274346 10.1152/physrev.2001.81.2.871

[B7] Bedard K. Jaquet V. Krause K. H. (2012). NOX5: from basic biology to signaling and disease. *Free Radic. Biol. Med.* 52 725–734. 10.1016/j.freeradbiomed.2011.11.023 22182486

[B8] Bedard K. Krause K. H. (2007). The NOX family of ROS-generating NADPH oxidases: physiology and pathophysiology. *Physiol. Rev.* 87 245–313. 10.1111/j.1471-4159.2011.07231.x 17237347

[B9] Bertoni A. Giuliano P. Galgani M. Rotoli D. Ulianich L. Adornetto A. (2011). Early and late events induced by polyQ-expanded proteins: identification of a common pathogenic property of polYQ-expanded proteins. *J. Biol. Chem.* 286 4727–4741. 10.1074/jbc.M110.156521 21115499 PMC3039353

[B10] Brown D. I. Griendling K. K. (2009). Nox proteins in signal transduction. *Free Radic. Biol. Med.* 47 1239–1253. 10.1016/j.freeradbiomed.2009.07.023 19628035 PMC2763943

[B11] Buntinx M. Vanderlocht J. Hellings N. Vandenabeele F. Lambrichts I. Raus J. (2003). Characterization of three human oligodendroglial cell lines as a model to study oligodendrocyte injury: morphology and oligodendrocyte-specific gene expression. *J. Neurocytol.* 32 25–38. 10.1023/A:1027324230923 14618099

[B12] Burdon R. H. Rice-Evans C. (1989). Free radicals and the regulation of mammalian cell proliferation. *Free Radic. Res. Commun.* 6 345–358. 10.3109/10715768909087918 2676744

[B13] Cavaliere F. Benito-Muñoz M. Panicker M. Matute C. (2013). NMDA modulates oligodendrocyte differentiation of subventricular zone cells through PKC activation. *Front. Cell. Neurosci.* 7:261 10.3389/fncel.2013.00261 PMC3866621 24391542

[B14] Cavaliere F. Urra O. Alberdi E. Matute C. (2012). Oligodendrocyte differentiation from adult multipotent stem cells is modulated by glutamate. *Cell Death Dis.* 3:e268 10.1038/cddis.2011.144 PMC3288341 22297298

[B15] Clemente D. Ortega M. C. Melero-Jerez C. de Castro F. (2013). The effect of glia-glia interactions on oligodendrocyte precursor cell biology during development and in demyelinating diseases. *Front. Cell. Neurosci.* 7:268 10.3389/fncel.2013.00268 PMC3868919 24391545

[B16] Cullen P. J. (2003). Calcium signalling: the ups and downs of protein kinase C. *Curr. Biol.* 13 R699–R701. 10.1016/j.cub.2003.08.041 13678606

[B17] D’Autréaux B. Toledano M. B. (2007). ROS as signalling molecules: mechanisms that generate specificity in ROS homeostasis. *Nat. Rev. Mol. Cell Biol.* 8 813–824. 10.1038/nrm2256 17848967

[B18] Damiano S. Fusco R. Morano A. De Mizio M. Paternò R. De Rosa A. (2012). Reactive oxygen species regulate the levels of dual oxidase (Duox1-2) in human neuroblastoma cells. *PLoS ONE* 7:e34405 10.1371/journal.pone.0034405 PMC3327694 22523549

[B19] Damiano S. Morano A. Ucci V. Accetta R. Mondola P. Paternò R. (2015). Dual oxidase 2 generated reactive oxygen species selectively mediate the induction of mucins by epidermal growth factor in enterocytes. *Int. J. Biochem. Cell Biol.* 60 8–18. 10.1016/j.biocel.2014.12.014 25562511

[B20] de la Monte S. M. Wands J. R. (2006). Molecular indices of oxidative stress and mitochondrial dysfunction occur early and often progress with severity of Alzheimer’s disease. *J. Alzheimers Dis.* 9 167–181. 16873964 10.3233/jad-2006-9209

[B21] di Penta A. Moreno B. Reix S. Fernandez-Diez B. Villanueva M. Errea O. (2013). Oxidative stress and proinflammatory cytokines contribute to demyelination and axonal damage in a cerebellar culture model of neuroinflammation. *PLoS ONE* 8:e54722 10.1371/journal.pone.0054722 PMC3576396 23431360

[B22] Dini L. Rotilio G. (1989). Electron microscopic evidence for endocytosis of superoxide dismutase by hepatocytes using protein-gold adducts. *Biochem. Biophys. Res. Commun.* 162 940–944. 10.1016/0006-291X(89)90763-8 2764947

[B23] Fontayne A. Dang P. M. Gougerot-Pocidalo M. A. El-Benna J. (2002). Phosphorylation of p47phox sites by PKC alpha, beta II, delta, and zeta: effect on binding to p22phox and on NADPH oxidase activation. *Biochemistry* 41 7743–7750. 10.1021/bi011953s 12056906

[B24] Franklin R. J. Ffrench-Constant C. (2008). Remyelination in the CNS: from biology to therapy. *Nat. Rev. Neurosci.* 9 839–855. 10.1038/nrn2480 18931697

[B25] Fyffe-Maricich S. L. Karlo J. C. Landreth G. E. Miller R. H. (2011). The ERK2 mitogen-activated protein kinase regulates the timing of oligodendrocyte differentiation. *J. Neurosci.* 31 843–850. 10.1523/jneurosci.3239-10.2011 21248107 PMC3568938

[B26] Gabrielli A. Svegliati S. Moroncini G. Pomponio G. Santillo M. Avvedimento E. V. (2008). Oxidative stress and the pathogenesis of scleroderma: the Murrell’s hypothesis revisited. *Semin. Immunopathol.* 30 329–337. 10.1007/s00281-008-0125-4 18548250

[B27] Ishii A. Furusho M. Bansal R. (2013). Sustained activation of ERK1/2 MAPK in oligodendrocytes and schwann cells enhances myelin growth and stimulates oligodendrocyte progenitor expansion. *J. Neurosci.* 33 175–186. 10.1523/jneurosci.4403-12.2013 23283332 PMC3711773

[B28] Jiang F. Zhang Y. Dusting G. J. (2011). NADPH oxidase-mediated redox signaling: roles in cellular stress response, stress tolerance, and tissue repair. *Pharmacol. Rev.* 63 218–242. 10.1124/pr.110.002980 21228261

[B29] Johnson J. R. Chu A. K. Sato-Bigbee C. (2000). Possible role of CREB in the stimulation of oligodendrocyte precursor cell proliferation by neurotrophin-3. *J. Neurochem.* 74 1409–1417. 10.1046/j.1471-4159.2000.0741409.x 10737596

[B30] Kageyama R. Ohtsuka T. Hatakeyama J. Ohsawa R. (2005). Roles of bHLH genes in neural stem cell differentiation. *Exp. Cell Res.* 306 343–348. 10.1016/j.yexcr.2005.03.015 15925590

[B31] Kameshwar-Rao A. S. Gil S. Richter-Landsberg C. Givol D. Yavin E. (1999). H2O2-induced apoptotic death in serum-deprived cultures of oligodendroglia origin is linked to cell differentiation. *J. Neurosci. Res.* 56 447–456. 10.1002/(SICI)1097-4547(19990601)56:53.0.CO;2-T 10369212

[B32] Katoh S. Mitsui Y. Kitani K. Suzuki T. (1997). Hyperoxia induces the differentiated neuronal phenotype of PC12 cells by producing reactive oxygen species. *Biochem. Biophys. Res. Commun.* 241 347–351. 10.1006/bbrc.1997.7514 9425274

[B33] Kennedy K. A. Sandiford S. D. Skerjanc I. S. Li S. S. (2012). Reactive oxygen species and the neuronal fate. *Cell. Mol. Life Sci.* 69 215–221. 10.1007/s00018-011-0807-2 21947442 PMC11114775

[B34] Lambeth J. D. (2004). NOX enzymes and the biology of reactive oxygen. *Nat. Rev. Immunol.* 4 181–189. 10.1038/nri1312 15039755

[B35] Li Q. Harraz M. M. Zhou W. Zhang L. N. Ding W. Zhang Y. (2006). Nox2 and Rac1 regulate H2O2-dependent recruitment of TRAF6 to endosomal interleukin-1 receptor complexes. *Mol. Cell. Biol.* 26 140–154. 10.1128/MCB.26.1.140-154.2006 16354686 PMC1317618

[B36] Lopez Juarez A. He D. Richard Lu Q. (2015). Oligodendrocyte progenitor programming and reprogramming: toward myelin regeneration. *Brain Res.* 1638 209–220. 10.1016/j.brainres.2015.10.051 26546966 PMC5119932

[B37] Lu Q. R. Sun T. Zhu Z. Ma N. Garcia M. Stiles C. D. (2002). Common developmental requirement for Olig function indicates a motor neuron/oligodendrocyte connection. *Cell* 109 75–86. 10.1016/S0092-8674(02)00678-5 11955448

[B38] Maes M. Galecki P. Chang Y. S. Berk M. (2011). A review on the oxidative and nitrosative stress (O&NS) pathways in major depression and their possible contribution to the (neuro)degenerative processes in that illness. *Prog. Neuropsychopharmacol. Biol. Psychiatry* 35 676–692. 10.1016/j.pnpbp.2010.05.004 20471444

[B39] McLaurin J. Trudel G. C. Shaw I. T. Antel J. P. Cashman N. R. (1995). A human glial hybrid cell line differentially expressing genes subserving oligodendrocyte and astrocyte phenotype. *J. Neurobiol.* 26 283–293. 10.1002/neu.480260212 7707048

[B40] Mondola P. Serù R. Santillo M. Damiano S. Bifulco M. Laezza C. (2002). Effect of Cu, Zn superoxide dismutase on cholesterol metabolism in human hepatocarcinoma (HepG2) cells. *Biochem. Biophys. Res. Commun.* 295 603–609. 10.1016/S0006-291X(02)00720-9 12099681

[B41] Morrison S. J. (2001). Neuronal potential and lineage determination by neural stem cells. *Curr. Opin. Cell Biol.* 13 666–672. 10.1016/S0955-0674(00)00269-6 11698181

[B42] Mukherjea D. Whitworth C. A. Nandish S. Dunaway G. A. Rybak L. P. Ramkumar V. (2006). Expression of the kidney injury molecule 1 in the rat cochlea and induction by cisplatin. *Neuroscience* 139 733–740. 10.1016/j.neuroscience.2005.12.044 16464536

[B43] Nissen C. Ciesielski-Treska J. Hertz L. Mandel P. (1973). Regulation of oxygen consumption in neuroblastoma cells: effects of differentiation and of potassium. *J. Neurochem.* 20 1029–1035. 10.1111/j.1471-4159.1973.tb00074.x 4697867

[B44] Noseworthy J. H. Lucchinetti C. Rodriguez M. Weinshenker B. G. (2000). Multiple sclerosis. *N. Engl. J. Med.* 343 938–952. 10.1056/NEJM200009283431307 11006371

[B45] Oakley F. D. Smith R. L. Engelhardt J. F. (2009). Lipid rafts and caveolin-1 coordinate interleukin-1beta (IL-1beta)-dependent activation of NFkappaB by controlling endocytosis of Nox2 and IL-1beta receptor 1 from the plasma membrane. *J. Biol. Chem.* 284 33255–33264. 10.1074/jbc.M109.042127 19801678 PMC2785168

[B46] Panchision D. M. (2009). The role of oxygen in regulating neural stem cells in development and disease. *J. Cell. Physiol.* 220 562–568. 10.1002/jcp.21812 19441077

[B47] Prosser B. L. Ward C. W. Lederer W. J. (2011). X-ROS signaling: rapid mechano-chemo transduction in heart. *Science* 333 1440–1445. 10.1126/science.1202768 21903813

[B48] Rao G. N. Berk B. C. (1992). Active oxygen species stimulate vascular smooth muscle cell growth and proto-oncogene expression. *Circ. Res.* 70 593–599. 10.1161/01.RES.70.3.593 1371430

[B49] Rogers T. B. Gaa S. T. Massey C. Dosemeci A. (1990). Protein Kinase C inhibits Ca^2+^ accumulation in cardiac sarcoplasmic reticulum. *J. Biol. Chem.* 265 4302–4308. 2155221

[B50] Samanta J. Kessler J. A. (2004). Interactions between ID and OLIG proteins mediate the inhibitory effects of BMP4 on oligodendroglial differentiation. *Development* 131 4131–4142. 10.1242/dev.01273 15280210

[B51] Santillo M. Colantuoni A. Mondola P. Guida B. Damiano S. (2015). NOX signaling in molecular cardiovascular mechanisms involved in the blood pressure homeostasis. *Front. Physiol.* 6:194 10.3389/fphys.2015.00194 PMC4493385 26217233

[B52] Sato-Bigbee C. Pal S. Chu A. K. (1999). Different neuroligands and signal transduction pathways stimulate CREB phosphorylation at specific developmental stages along oligodendrocyte differentiation. *J. Neurochem.* 72 139–147. 10.1046/j.1471-4159.1999.0720139.x 9886064

[B53] Sauer H. Wartenberg M. Hescheler J. (2001). Reactive oxygen species as intracellular messengers during cell growth and differentiation. *Cell Physiol. Biochem.* 11 173–186. 10.1159/000047804 11509825

[B54] Serù R. Mondola P. Damiano S. Svegliati S. Agnese S. Avvedimento E. V. (2004). HaRas activates the NADPH oxidase complex in human neuroblastoma cells via extracellular signal-regulated kinase 1/2 pathway. *J. Neurochem.* 91 613–622. 10.1111/j.1471-4159.2004.02754.x 15485492

[B55] Smith J. Ladi E. Mayer-Proschel M. Noble M. (2000). Redox state is a central modulator of the balance between self-renewal and differentiation in a dividing glial precursor cell. *Proc. Natl. Acad. Sci. U.S.A.* 97 10032–10037. 10.1073/pnas.170209797 10944195 PMC27662

[B56] Svegliati S. Cancello R. Sambo P. Luchetti M. Paroncini P. Orlandini G. (2005). PDGF and reactive oxygen species (ROS) regulate Ras protein levels in primary human fibroblasts via ERK1/2: amplification of ROS and Ras in systemic sclerosis fibroblasts. *J. Biol. Chem.* 280 36474–36482. 10.1074/jbc.M502851200 16081426

[B57] Syed Y. A. Baer A. Hofer M. P. González G. A. Rundle J. Myrta S. (2013). Inhibition of phosphodiesterase-4 promotes oligodendrocyte precursor cell differentiation and enhances CNS remyelination. *EMBO Mol. Med.* 5 1918–1934. 10.1002/emmm.201303123 24293318 PMC3914530

[B58] Thorburne S. K. Juurlink B. H. (1996). Low glutathione and high iron govern the susceptibility of oligodendroglial precursors to oxidative stress. *J. Neurochem.* 67 1014–1022. 10.1046/j.1471-4159.1996.67031014.x 8752107

[B59] Ueno N. Takeya R. Miyano K. Kikuchi H. Sumimoto H. (2005). The NADPH oxidase Nox3 constitutively produces superoxide in a p22phox-dependent manner: its regulation by oxidase organizers and activators. *J. Biol. Chem.* 280 23328–23339. 10.1074/jbc.M414548200 15824103

[B60] Wang Y. Chen F. Le B. Stepp D. W. Fulton D. J. (2014). Impact of Nox5 polymorphisms on basal and stimulus-dependent ROS generation. *PLoS ONE* 9:e100102 10.1371/journal.pone.0100102 PMC4081039 24992705

[B61] Zhang Y. Guo T. B. Lu H. (2013). Promoting remyelination for the treatment of multiple sclerosis: opportunities and challenges. *Neurosci. Bull.* 29 144–154. 10.1007/s12264-013-1317-z 23558587 PMC5561877

