# Supplementary material for: Reactive Oxygen Species Derived from NOX3 and NOX5 Drive Differentiation of Human Oligodendrocytes
Source: Front Cell Neurosci. 2016 Jun 2;10:146. doi: 10.3389/fncel.2016.00146 (PMC4889614; doi:10.3389/fncel.2016.00146)
Supplement: Supplementary file 1 [file Image_1.PDF]

Supplementary Material

Reactive oxygen species derived from NOX3 and NOX5 drive differentiation of human oligodendrocytes

Roberta Accetta, Simona Damiano, Annalisa Morano, Paolo Mondola, Roberto Paternò, Enrico Vittorio Avvedimento and Mariarosaria Santillo*

*** Correspondence:** Corresponding Author: marsanti@unina.it

1 Supplementary Figures

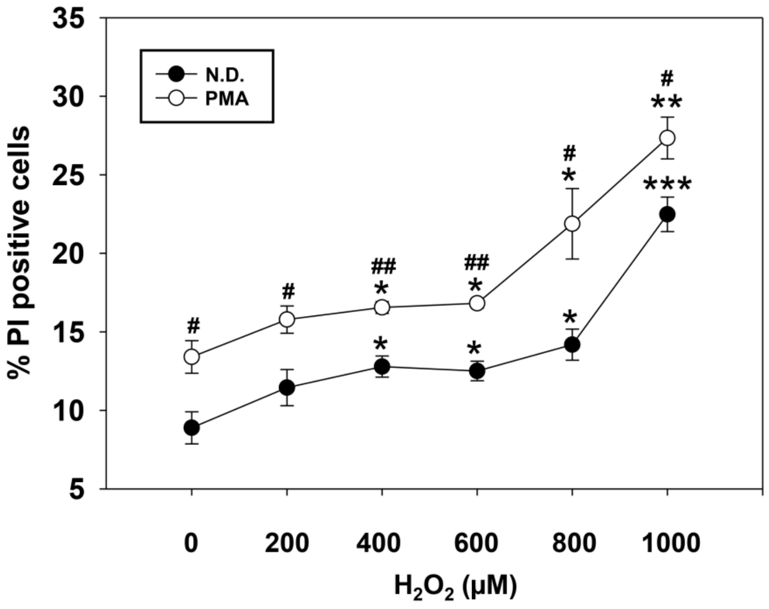

1.1 Supplementary Figure 1: Dose-dependent effect of H_2O_2 on cell viability in the presence of serum.

MO3-13 cells, plated in 35mm Petri dishes, were differentiated in FBS-free DMEM supplemented with 100 nM of PMA for 4 days. Not differentiated (N.D.), growing in complete medium, and differentiated (PMA) MO3-13 cells were stimulated for 18h with increasing doses of H_2O_2 (200, 400, 600, 800, 1000µM) in the presence of 10% FBS and cell viability was evaluated by cytofluorimetry after Propidium Iodide (PI) staining. After trypsinization and wash in PBS, the cells were resuspended in 500µl of PBS and 1µg/ml of PI was added before the flow cytometric analysis of PI-

positive cells performed with a FCSscan apparatus (Becton-Dickinson). Data were analyzed using WinMDI 2.9 software. The graphs show the mean +/- SEM values relative to control of three independent experiments. *p< 0.05, **p<0.01, ***p<0.001 vs Control; #p< 0.05, ##p< 0.01 vs Corresponding dose.

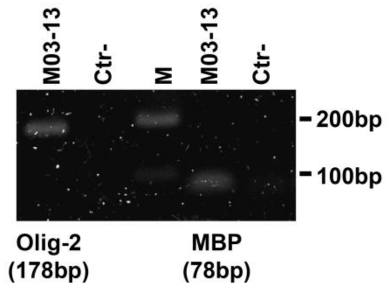

1.2 Supplementary Figure 2: Expression of the specific oligodendrocyte markers Olig-2 and MBP in MO3-13 cells.

Total RNA was extracted, using TRIzol reagent according to the protocol provided by the manufacturer (Sigma-Aldrich), and reverse transcribed (1 µg) using Transcriptor First Strand cDNA Synthesis Kit (Roche Applied Science) by oligo-dT primers for 30 min at 55 °C in a 20 µl reaction volume; then it was analyzed by PCR with specific primers to Olig-2 (F, CCA GAG CCC GAT GAC CTT TTT; R, CAC TGC CTC CTA GCT TGT C)or MBP(F, CTC CAT CGG GCG CTT CTT TG; R, CGG GTG GTG TGA GTC CTT GT). Qualitative PCR was performed using Hot Master Taq DNA Polymerase (5PRIME) in 20µl final volume containing 0.2 mM dNTP, 0.2 µM of the specific primers and 100 ng of sample cDNA. The PCR conditions used were 94°C 2 min, (94°C 30 sec, 60°C 30 sec, 70°C 30 sec) and 70°C 5 min in GeneAmp PCR Sysyem 9700 (Applied Biosystem Inc, USA). The reactions were carried out at different cycles (30-35). Ctr-, negative control; M, marker.

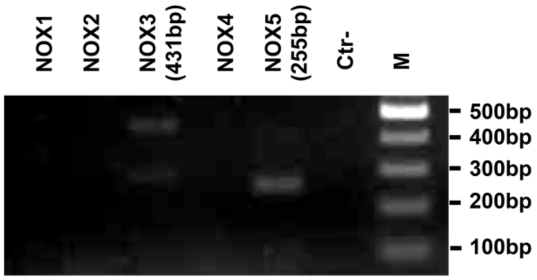

1.3 Supplementary Figure 3: Identification of NOXs expressed in MO3-13 cells.

Total RNA was extracted, using TRIzol reagent according to the protocol provided by the manufacturer (Sigma-Aldrich), and reverse transcribed (1 µg) using Transcriptor First Strand cDNA

Synthesis Kit (Roche Applied Science) by oligo-dT primers for 30 min at 55 °C in a 20 µl reaction volume; then it was analyzed by PCR with specific primers to NOX1(F, TTA ACA GCA CGC TGA TCC TG; R, CAC TCC AGT GAG ACC AGC AA), NOX2 (F, GGA GTT TCA AGA TGC GTG GAA ACT A; R, GCC AGA CTC AGA GTT GGA GAT GCT), NOX3(F, CCA GGG CAG TAC ATC TTG GT; R, CCG TGT TTC CAG GGA GAG TA), NOX4 (F, GCT TAC CTC CGA GGA TCA CA; R, CGG GAG GGT GGG TAT CTA A) and NOX5(F, ATC AAG CGG CCC CCT TTT TTT CAC; R, CTC ATT GTC ACA CTC CTC GAC AGC). Qualitative PCR was performed using Hot Master Taq DNA Polymerase (5PRIME) in 20µl final volume containing 0.2 mM dNTP, 0.2 µM of the specific primers and 100 ng of sample cDNA. The PCR conditions used were 94°C 2 min, (94°C 30 sec, 60°C 30 sec, 70°C 30 sec) and 70°C 5 min in GeneAmp PCR Sysyem 9700 (Applied Biosystem Inc, USA). The reactions were carried out at different cycles (30-35). Ctr-, negative control; M, marker.
